# Polymersome-based protein drug delivery – quo vadis?†

**DOI:** 10.1039/d2cs00106c

**Published:** 2022-12-20

**Authors:** Micael G. Gouveia, Justus P. Wesseler, Jobbe Ramaekers, Christoph Weder, Philip B. V. Scholten, Nico Bruns

**Affiliations:** a Department of Pure and Applied Chemistry, University of Strathclyde Thomas Graham Building 295 Cathedral Street Glasgow G1 1XL UK; b Adolphe Merkle Institute Chemin des Verdiers 4 1700 Fribourg Switzerland philipscholten@gmail.com; c Department of Chemistry, Technical University of Darmstadt Alarich-Weiss-Straße 4 64287 Darmstadt Germany nico.bruns@tu-darmstadt.de

## Abstract

Protein-based therapeutics are an attractive alternative to established therapeutic approaches and represent one of the fastest growing families of drugs. While many of these proteins can be delivered using established formulations, the intrinsic sensitivity of proteins to denaturation sometimes calls for a protective carrier to allow administration. Historically, lipid-based self-assembled structures, notably liposomes, have performed this function. After the discovery of polymersome-based targeted drug-delivery systems, which offer manifold advantages over lipid-based structures, the scientific community expected that such systems would take the therapeutic world by storm. However, no polymersome formulations have been commercialised. In this review article, we discuss key obstacles for the sluggish translation of polymersome-based protein nanocarriers into approved pharmaceuticals, which include limitations imparted by the use of non-degradable polymers, the intricacies of polymersome production methods, and the complexity of the *in vivo* journey of polymersomes across various biological barriers. Considering this complex subject from a polymer chemist's point of view, we highlight key areas that are worthy to explore in order to advance polymersomes to a level at which clinical trials become worthwhile and translation into pharmaceutical and nanomedical applications is realistic.

## Abstract 2

Block copolymer vesicles are well suited as nano-sized drug delivery vehicles for therapeutic proteins. However, they have not reached the clinic yet. Why? The review discusses opportunities and obstacles of polymersome-based protein drug delivery.

## Graphical abstract

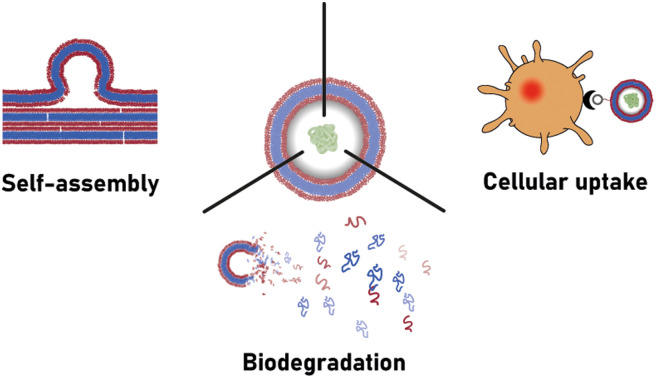

## 1. Introduction

**Fig. 1:**
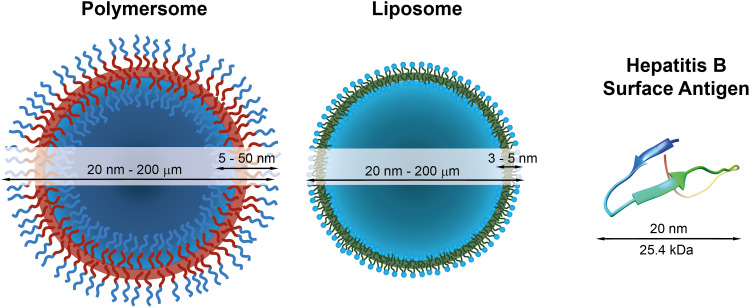
Schematic representations of a polymersome, a liposome, and the hepatitis surface antigen B, as a typical example of a therapeutically useful protein,^22^ with indications of typical diameters and membrane thicknesses. Protein graphic made with UCSF Chimera.^23^

The COVID-19 pandemic has highlighted the dangers of new viruses and diseases that can spread within a few months across the globe. Fortunately, the response was in this case swift, and several novel vaccines have rapidly become available or advanced in clinical trials. The most effective vaccines that are now being administered to a global population are based on messenger ribonucleic acid (mRNA) and rely on lipid nanoparticles to encapsulate, protect, and deliver the mRNA into the immune cells.^1,2^ This is a very effective strategy of protecting sensitive cargo from denaturation and other degrading factors inside the body. Lipid nanoparticles are structurally similar to lipid vesicles, so-called liposomes, which were first discovered in the 1960s^3^ and have proven to be very useful as cargo carriers for a variety of therapeutics. Nonetheless, the lipid bilayer of liposomes and lipid nanoparticles is oftentimes only stable at low temperatures for extended periods of time and, in the case of some of the mRNA vaccines, requires cooling to unpractically low temperatures (−70 °C) in order to remain intact and to keep the formulation therapeutically active.^4^ Polymersomes, which consist of self-assembled amphiphilic block copolymers, are promising alternatives to liposomes, because of (i) the chemical versatility of the polymer structure, (ii) the ease of synthesis, (iii) the possibility to add further functions, such as stimuli-responsiveness, and (iv) the increased stability of the self-assembled polymersome structures compared to liposomes and lipid nanoparticles.^4–17^ Similar to liposomes, which are self-assembled vesicles of amphiphilic lipids, polymersomes are vesicles that are composed of amphiphilic macromolecules, typically block copolymers. The amphiphilic polymer molecules form a bilayer membrane that encloses a large hydrophilic lumen with a diameter ranging from 20 nm to several hundreds of micrometres (Fig. 1). This lumen is large enough to accommodate macromolecular cargo such as mRNA and proteins, whose typical volume ranges from 4000 nm^3^ to 4 000 000 μm^3^.^4^ Commonly used hydrophobic blocks in polymersome-forming block copolymers are poly((meth)acrylates) and polydimethylsiloxane (PDMS), while the hydrophilic polymer of choice is often poly(ethylene glycol) (PEG), hydrophilic poly(meth)acrylates or poly(2-methyl-2-oxazoline). Further details on the individual blocks that are typically used can be found in Section 2, while previous reviews detail the synthetic methods.^16–20^ The wealth of polymer structures available and the ease with which these can be synthesised lead to a versatility that is not as easily achievable with lipids. On the one hand, the lipid structure consists of much smaller building blocks with typically only between 10 and 30 carbon atoms (*c.f*. polymers > 100 C atoms), thus restricting the possibilities of changing and functionalising these building blocks. Another important aspect of polymersomes is that the high molecular weight of the hydrophilic and hydrophobic segments greatly increases the energy barrier for a polymer to leave a self-assembled structure or to rearrange itself completely. Therefore, polymersomes are little dynamic and can be stable at room temperature for months. By contrast, liposomes and lipid nanoparticles are typically highly dynamic under ambient conditions and typically need to be stored at low temperature in order to remain in the originally assembled state. However, a main disadvantage of polymers and their self-assembled structures is that the toxicological information of the building blocks is too often not known and needs to be generated before approval by regulatory bodies, such as the Federal Drug Administration (FDA) in the US or the European Medical Agency (EMA). Contrary to polymers, the natural abundance of lipids and their established use in biotechnology and pharmaceutics means that this information is readily available for them. In addition, their natural abundance means that pathways to their biodegradation exists,^21^ which is not always the case for polymers.

Independent of the delivery system used, the protection of bioactive molecules and their delivery to discrete and specific sites in the body are the main functions of therapeutic delivery systems, but these remain in many cases elusive. Proteins play a crucial role in all living beings and bear a great potential to cure a variety of diseases.^24–27^ The use of therapeutic proteins in novel drugs is of particular interest due to their many intrinsic advantages, such as high specificity to defined biological processes, biodegradability, and (often) natural availability. Moreover, proteins can offer therapeutic effects that cannot be achieved with small molecule drugs and are, *e.g.*, promising candidates to combat and prevent multi-resistant bacteria. The ability to accurately programme the protein structure using novel techniques, such as CRISPR, is a further advantage and has led to increased research in the pharmaceutical industry.

The most prominent therapeutic protein currently in use forms the basis of the hepatitis B vaccine, which has been available since 1982 and was given to 84% of infants worldwide in 2015.^28^ It consists of the hepatitis B surface antigen (Fig. 1), which triggers the body's immune response to this pathogen. The World Health Organisation estimates that 14.2 million infections in children under the age of five were prevented because of the vaccination activities since the 1980s.^29^ Another widespread example is insulin, which is used to regulate the blood sugar level in patients with sugar insufficiency (diabetes). Other therapeutic proteins include proteins and therapeutic antibodies to treat a variety of severe diseases,^30,31^ such as HIV^32^ and cancer.^33^ These examples highlight the different medical applications of therapeutic proteins and antibodies and exemplify their power to treat or prevent specific illnesses and improve our daily lives.

Nonetheless, proteins remain difficult to administer to specific sites in the body, as a result of their sensitivity to denaturation and clearance from the blood stream.^34^ Moreover, the uptake of proteins through the hydrophobic cell membrane is hampered because of their high molecular weight and their hydrophilicity.^35^ As a result, delivery vehicles are necessary to provide a protective shield against denaturation and to hide proteins from the clearing mechanisms of the blood stream.^36,37^ Such carriers must provide a sufficiently large hydrophilic pocket that allows an effective encapsulation of the protein. The most suited candidates for such protein delivery systems are liposomes and polymersomes (Fig. 1).

The successful encapsulation of proteins can be achieved without chemical modifications on their surface. In addition to the hydrophilic cargo in their lumen, liposomes and polymersomes can simultaneously carry hydrophobic molecules in the hydrophobic membrane.^38^ Furthermore, the corona of the capsules can be easily decorated with targeting molecules that are specific to certain receptors, thus allowing for site specific and targeted drug delivery, and thereby mimicking a viral-like delivery of biomolecules.

Surprisingly, in 2022 there were no polymersome protein delivery systems available on the market, or in advanced stages of clinical trials.^5,39^ Several start-up companies provide the know-how and the polymersomes themselves for successful internalisation into cells,^40,41^ but to the best of our knowledge, this has not been translated into commercialised therapeutics delivering actual drugs or proteins. On the other hand, three protein-loaded liposome systems are commercially available as vaccines against hepatitis C and the flu, as well as a treatment for neonatal respiratory distress syndrome.^35^ Four other protein-loaded liposomal systems are at different phases of clinical trials and target a variety of diseases such as diabetes and skin cancer.^35^

A review of the scientific literature reveals that a multitude of studies are devoted to developing polymersome delivery systems loaded with proteins as therapeutics for a variety of diseases.^9,15,36,42^ However, the lack of polymersome systems in clinical trials begs the question: *Why are polymersomes lagging behind liposomes?* An important aspect is the origin of the encapsulating material. While liposomes are most often based on naturally abundant phospholipids, such as naturally occurring phosphatidylcholine, amphiphilic block copolymers are almost exclusively made synthetically. This has severe implications for the approval of polymersomes in clinical use, as the toxicity, cellular uptake and cellular exit, as well as degradation of these novel polymer structures in the body have to be established prior to approval. Moreover, the inherent molecular weight distribution of polymer chains makes regulatory approval more difficult.

Their more recent discovery is perhaps the most important factor that has held back polymersomes over liposomes in drug-delivery applications: while liposomes have been known and were developed since the 1970s,^43–47^ polymersomes were first described in the late 1990s^48,49^ and have therefore only been investigated as drug delivery vehicles over the last two decades.^5^ This is reflected in the number of publications concerning liposomes and polymersomes in drug delivery, around 24 000^50^ and 5400^51^ journal articles, respectively. Moreover, in the light of the long and cumbersome road towards approval of drug-delivery systems, with an estimated average of around twenty years, the first successful clinical trials of polymersomes cannot realistically be expected before 2025, unless unexpected developments, such as a global pandemic, greatly accelerate research and development of these drug-delivery systems. The clinical experience and knowledge gained in the context of liposome-based systems are, however, a great resource for the polymersome community to draw on, as the big question remains: Can polymersomes be developed that overcome the shortcomings of liposomes and can highly specific, stable, and potent protein delivery vehicles be created? We herein address, with a focus on the delivery of proteins, some of the critical issues that remain to be addressed, namely degradability, scalable and reproducible polymersome preparation, and cellular uptake and release. Drawing parallels from the developments of liposomes, we outline potential solutions and unexplored areas, which are promising opportunities within the polymersome field and warrant further research.

## 2. Choice of polymer type

**Fig. 2:**
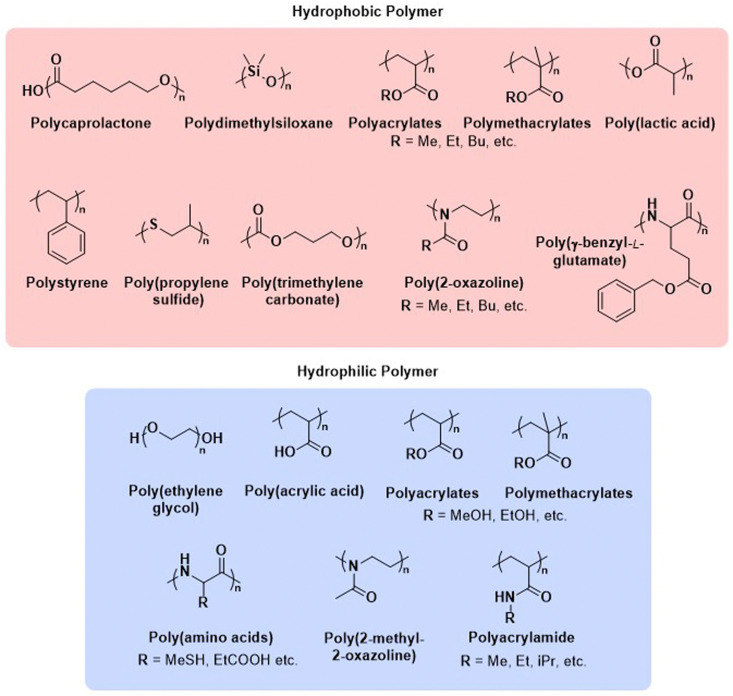
Chemical structures of common polymers used as the hydrophobic and hydrophilic blocks of polymersome-forming block copolymers.

Before we summarise the state-of-the-art and discuss future directions of the polymersome field, the most widely employed processes and workflows for polymersome preparation are briefly reviewed. Prior to any assembly process, the choice of the block copolymer is of outmost importance, as this determines many factors of the final polymersome, including the membrane thickness, stability, and biological response in the body. A vast library of polymers from which block copolymers can be constructed, almost without limits, is available. An overview of the most common hydrophobic and hydrophilic blocks is given in Fig. 2. The most commonly used polymers for the hydrophobic block are poly(caprolactone) (PCL), poly(acrylates) and poly(methacrylates) with varying substituents, and poly(lactic acid), while the hydrophilic blocks are most commonly based on poly(ethylene glycol) (PEG) and poly(amino acids). The synthetic methods used to access these building blocks and/or the final block copolymers range from ring-opening polymerisation to reversible deactivation radical polymerisations and the preparation of such materials has been extensively discussed in previous reviews.^16–20^ Suitable polymer architectures not only include diblock copolymers but also macromolecules featuring three, or more, blocks. Of course, the structure has a pronounced effect on the self-assembly process and the final properties of the polymersomes. Another critical aspect of these block copolymers that determines their final self-assembled shape is the ratio of the molecular weights of the hydrophilic and hydrophobic components, and the interplay of free energy and kinetic contributions determines the final structure.^52,53^ As a rule of thumb, block copolymers with a hydrophilic:hydrophobic molecular weight ratio of 1 : 1 typically assemble into micelles, while a ratio of 1 : 3 lead to polymersome formation.^4,54^ These ratios are not precise design elements – other self-assembled structures can form – and there are often compositional ranges in which different assemblies coexist. More exact predictions can be made based on the packing parameter and the volume fraction of each polymer block.^4,53,55^ However, the packing parameter can usually only roughly be determined for block copolymers.

Important aspects that set polymersomes apart from liposomes are their synthetic nature and their oftentimes poor biodegradability, which is obviously of concern in drug delivery applications. It is therefore crucial for polymersomes to be biodegradable and biocompatible, with well-defined and favourable clearance mechanisms akin to liposomes. The thick and stable membrane of polymersomes allows polymersomes to efficiently protect their encapsulated cargo, evade the reticuloendothelial system (RES), and enable long circulation times.^4,56,57^ However, this also means that polymersomes are prone to linger in certain organs or have insufficient drug diffusion once they have reached their target site.^58^ Toxicity issues due to accumulation, posing severe limitations on dosage, are therefore a key challenge to overcome. Consequently, considerable efforts have been devoted to designing degradable and biocompatible polymersomes, generating polymersomes that degrade into smaller oligomers or monomers for rapid renal clearance. To create polymersomes that are potentially useful in medical applications, the impact of the polymer block choice must be thoroughly considered, as even though a polymer may be biodegradable, its degradation products could still pose a risk of toxicity.^59^ If degradation (or disassembly into unimeric chains) can be tailored to occur upon local environmental changes, it may additionally act as a means of controlled release.^60^ These aspects of polymersome degradation are explored below.

## 3. Biodegradable polymersomes

Studies which account for *in vitro* cell viability and *in vivo* effect and fate of polymersomes are generally carried out with polymers whose biodegradability and biocompatibility are well-established, such as poly(dl-lactide), poly(trimethylene carbonate), and poly(caprolactone).^61–66^ More recent publications on polymersomes involving less-frequently studied polymers or polymers bearing additional functional species have little biodegradability and biocompatibility data available, hence requiring extensive work to establish their suitability for medicinal purposes.^5,67–70^

Most biodegradable polymers for clinical applications degrade *via* hydrolysis and/or enzymatic processes.^71^ The hydrolytic susceptibility of block copolymers under various physiological conditions is tuneable and therefore already allows for informed control of degradation. Key design parameters include the choice of the hydrolysable group,^72^ the polymer crystallinity,^73–75^ and the initial molecular weight.^71^ Localised environmental factors, such as the pH or the presence of specific enzymes, may also contribute to rapid polymer hydrolysis and can be exploited for controlled cargo release. In cases where polymersomes are desired to remain inside the body for prolonged periods of time, it is important to avoid premature degradation and unwanted immune responses, which can be achieved by selecting polymer species that are biocompatible and offer slow biodegradation over the course of weeks or months. As biodistribution and specific cellular uptake are key considerations for therapeutic polymersomes, the intracellular environment has also been used as a means of triggering degradation of polymersomes, through the incorporation of specific enzyme cleavable units.^76–80^ Ultimately, polymersome biodegradation is an important factor that influences toxicity limits and biodistribution of the encapsulated cargo.

Herein, we highlight selected examples of biodegradable polymersomes reported in the literature with potential for medicinal application, discussing the various degradation pathways and means of harnessing these beneficially. The understanding of polymersome biodegradation is important as it affects site selective delivery of therapeutics and pharmacokinetics of both polymersome and cargo.

**Scheme 1:**
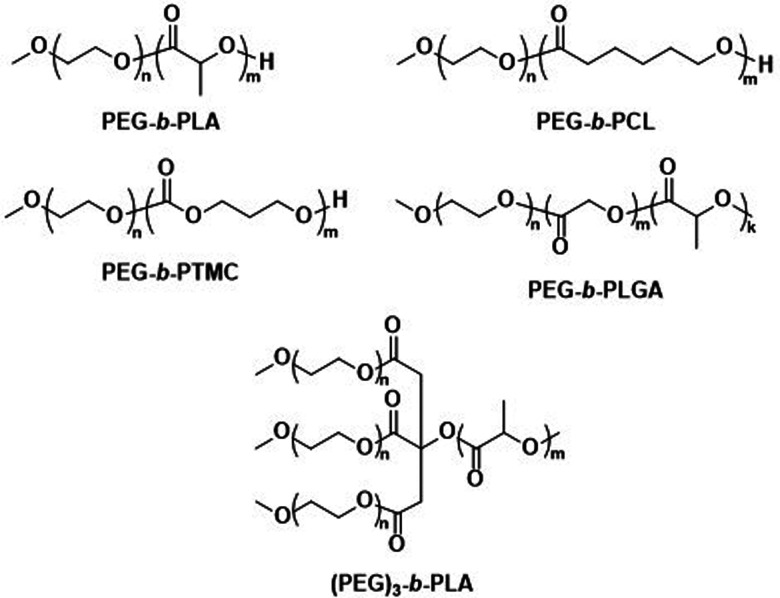
Chemical structures of biodegradable block copolymers based on PEG as the hydrophilic block and biodegradable hydrophobic blocks. PEG-*b*-PLA, PEG-*b*-PCL and PEG-*b*-PTMC were used in initial report on biodegradable polymersomes (Feijen and coworkers^81^). PEG-*b*-PLGA polymersomes have documented success in preclinical trials as DOX delivery agents (Alibolandi *et al.*^83^). (PEG)_3_-*b*-PLA copolymer can generate polymersomes with a high mol% of biodegradable blocks (Kumar and cowokers^84,85^).

The first report on biodegradable polymersomes by Feijen *et al.* appeared almost two decades ago,^81^ who assembled amphiphilic block copolymers consisting of the biodegradable hydrophobic polymers poly(d,l-lactide) (PLA or PDLLA), poly(ε-caprolactone) (PCL), or poly(trimethylene carbonate) (PTMC) and PEG as the hydrophilic block (Scheme 1).^81^ For polymersomes based on well-known biodegradable blocks, the rate of biodegradation in other studies involving free polymer chains, films, or nanoparticles can often be used to extrapolate this data.^82^

PDLLA has been widely studied as a biodegradable polymer for biomedical applications.^82^ Its hydrolytic degradation in PBS buffer is very slow and proceeds over the course of years,^86^ while enzymatic degradation (*via* proteases, lipases, *etc.*) is much faster and complete within weeks.^87,88^ The copolymer poly(lactide-*co*-glycolic acid) (PLGA) has a higher hydrolysis rate (non-enzymatic) than pure PDLLA, and the degradation rate increases with the glycolide content (approx. 4–5 months for full degradation in PBS, pH 7.4). PCL is a semi-crystalline polymer which, similar to PDLLA, undergoes slow hydrolytic degradation (years in PBS) yet rapidly degrades in the presence of enzymes such as *Pseudomonas* lipase (days).^89^ PTMC is a non-crystalline polymer, resistant to non-enzymatic hydrolysis and therefore biodegrades slower compared to aliphatic polyesters (PDLLA, PCL, *etc.*).^90,91^ This slower degradation has led to suggestions that PTMC based polymersomes are better suited for prolonged release of drugs.^92^

**Fig. 3:**
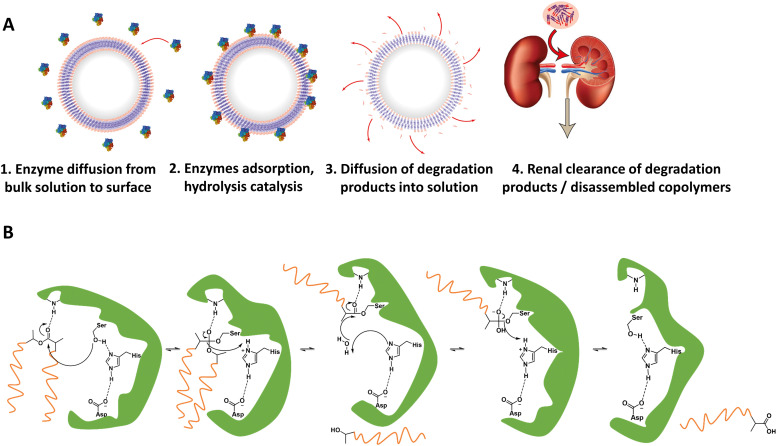
(A) Representation of enzymatic degradation process of polymer block(s) in polymersomes. (1 and 2) Initial diffusion of enzymes in the surrounding media to the polymersome membrane followed by adsorption of the enzyme onto the polymersome. Biodegradable block(s) may now act as substrates for the enzymes, whereupon the catalytic hydrolysis can take place. (3) Biodegradation breaks apart block copolymers in membrane, and degradation products start to separate from polymersome membrane. (4) Chain fragments are small enough for excretion. (B) Example mechanism of enzymatically catalyzed polymer degradation: an esterases’ catalytic triad in its active site catalyzing the hydrolysis of a PLA ester bond.

For *in vivo* polymersome degradation, factors affecting enzymatic degradation are a key consideration (Fig. 3). Enzymatic adsorption on the polymersomes and hydrolysis of polymers are dependent on their physicochemical properties, such as the molecular weight of the polymers, crystallinity, chemical composition, and surface area.^93^ The possibility to combine biodegradable copolymers, *a priori* in any combination and ratio, allows one to tune the degradation rate *in vivo* over a wide range.^93^ Another important feature relevant to polymersome biodegradation is the preferential hydrolysis of ester bonds connecting hydrophobic polyesters to PEG.^82^ This process generally causes rapid disassembly, cargo release, and/or rapid excretion. Care should therefore be taken when designing polymersomes as unwanted cleavage between blocks may compromise shelf-life and control of cargo release *in vivo*. While the initial studies of biodegradable polymersomes did not take protein delivery into account, their shelf life and resistance to hydrolysis was investigated. The limited stability – 3 months when stored in water – may raise practical concerns, notably with respect to product shelf life, although some current lipid/viral based gene delivery systems require cooling (down to −80 °C) with similar shelf-lives. Lyophilisation could improve shelf life for hydrolytically sensitive polymersomes, although other issues such as membrane rupture, deformation of vesicles or their aggregation, may arise.^94–96^ Further studies by Feijen *et al.* on polymersomes based on PEG-*b*-PDLLA and PEG-*b*-PCL block copolymers showcase differences in the stability of hydrolysable blocks in phosphate-buffered saline (PBS) at 4 °C and 37 °C.^61^ The degradation rates were monitored *via* dynamic light scattering (DLS), showing dependence on the polymer structure and crystallinity. These findings are supported by other studies, which highlight that the degree of crystallinity in hydrolysable polymers has a significant effect on hydrolysis rate.^75^

Polymersomes based on block copolymers of PEG and a biodegradable hydrophobic block have since been investigated as potential drug delivery systems, including comparisons to liposomes. Alibolandi *et al.* performed thorough preclinical tests on PEG-*b*-poly(d,l-lactide-*co*-glycolic acid) (PEG-*b*-PLGA) (Scheme 1) polymersomes for doxorubicin (DOX) encapsulation and compared these to a mimic of the liposomal formulation Doxil.^83^ These *in vivo* studies revealed a lower maximum tolerated dose than the liposomal reference formulation, but at the same time a three-fold higher therapeutic efficacy of the polymersomes was observed, leading to an overall lower required dose to achieve tumour growth suppression.^83^ Importantly, no histopathological changes in the lung, kidney, and liver were observed after administration of multiple doses. By contrast, liposomes showed atrophy in the liver, which provides strong evidence that polymersomes may contend liposomes in clinical trials and further developments are warranted. Kumar *et al.* reported on three-armed (PEG)_3_-*b*-PLA copolymers (Scheme 1) joined through a citric acid linker.^84,85^ These copolymers contain PLA as a biodegradable hydrophobic block and are attractive because they form polymersomes even if the content of the non-biodegradable hydrophilic PEG block is as low as 10 mol%. Such (PEG)_3_-*b*-PLA polymersomes were loaded with DOX and their pharmacokinetics and toxicity profiles were thoroughly studied and compared to LipoDox™ in treating mammary carcinoma.^85^ In spite of positive data, in particular rapid and specific DOX concentration in tumours, further advancements of clinical studies of (PEG)_3_-*b*-PLA polymersomes have not yet been reported.

### 3.1. Polypeptide and biopolymer biodegradable polymersomes

The biodegradable polymersomes described so far consist of biodegradable hydrophobic blocks conjugated to hydrophilic PEG blocks. While PEG is often selected as a biocompatible hydrophilic block with known stealth properties to increase the blood circulation time of nanoparticles, it is not biodegradable and the complete clearance of PEG chains may take several months.^97,98^ Moreover, certain studies have raised concerns over its toxicology and immune response, especially in consecutive administrations, see discussion in Section 6.1.^99–101^

**Scheme 2:**
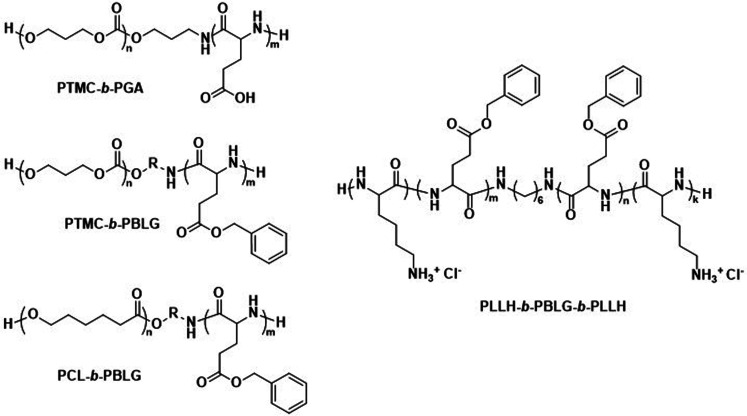
Chemical structures of biodegradable block copolymers using poly(amino acids) (PAmA) as hydrophobic and/or hydrophilic blocks. The polymersomes formed from these blocks are fully enzymatically biodegradable. Assemblies containing PTMC blocks degrade faster than assemblies containing PCL blocks. PTMC-*b*-PGA based polymersomes change size in response to shifts in pH/ionic strength (Lecommandoux and coworkers^104^). PLLH-*b*-PBLG-*b*-PLLH block copolymers favor polymersome formation due to the rod-like structure of the central PBLG block. The phenyl rings of PBLG enable high loading of paclitaxel through π–π interactions. When loaded with DOX, PLLH-*b*-PBLG-*b*-PLLH polymersomes show similar efficacy to Myocet (commercially available DOX loaded liposome formulation) (Iatrou *et al.*^111^).

Consequently, alternative hydrophilic biodegradable polymers have been explored as polar building blocks for polymersomes, including poly(amino acids) (PAmAs) and polysaccharides. PAmAs are interesting as PEG alternatives, because they are fully degradable by enzymes and exhibit a low toxicity.^102,103^ Representative examples were reported by Lecommandoux *et al.* who investigated PCL-*block*-poly(γ-benzyl-l-glutamate) (PCL-*b*-PBLG), PTMC-*block*-poly(l-glutamic acid) (PTMC-*b*-PGA), and PTMC-*block*-PBLG (PTMC-*b*-PBLG) block copolymers in the form of either nanoparticles or polymersomes (Scheme 2).^104,105^ While non-enzymatic hydrolytic degradation of both PTMC and PCL is very slow, enzymatic hydrolysis *via* lipases (*e.g.*, *Pseudomonas* lipase) proceeded rapidly within 40 hours. Differences in degradation rates of PTMC-*b*-PBLG and PCL-*b*-PBLG nanoparticles *via Pseudomonas* lipase were observed. The observation that the PTMC-*b*-PBLG nanoparticles degraded faster was explained by the fact that the amorphous PTMC is more accessible to the enzyme than the semi-crystalline PCL, which highlights that degradation rate is not only affected by the chemical structure of the polymer but also its morphology. Hydrophilic PGA is hydrolytically stable, yet susceptible to degradation by certain enzymes, such as cathepsin B, which is overexpressed in tumour cells.^106^ PTMC-*b*-PGA polymersomes were found to be highly resistant towards water permeability once assembled, indicating that these polymersomes are robust carriers of therapeutic payloads, only undergoing significant degradation and release upon exposure to specific enzymes.^106^ Further studies using poly(amino acids) to generate polymersomes have established their biocompatibility and improved therapeutic delivery *via* enzyme specific degradation.^107–110^ Iatrou *et al.* reported polymersomes based on an ABA triblock copolymer consisting entirely of amino acids – poly(l-lysine hydrochloride)-*b*-PBLG-*b*-poly(l-lysine hydrochloride) (PLLH-*b*-PBLG-*b*-PLLH) (Scheme 2) – that were loaded with DOX. The authors reported a similar efficacy in inhibiting human pancreatic cell line growth as observed for the liposomal cancer medicine Myocet in *in vitro* experiments.^111^*In vivo* experiments in which empty PLLH-*b*-PBLG-*b*-PLLH polymersomes were administered to mice confirmed their biocompatibility, showing no signs of toxicity at concentrations of up to 200 mg kg^−1^.^111^ This example highlights that PAmAs can fulfil the role of both hydrophobic and hydrophilic blocks and generally lead to non-toxic degradation products, making them an attractive alternative to PEG. The specific degradation of polymersomes through enzymatic processes is also possible if adequate peptide sequence linkers are used between the two copolymer blocks; this approach is discussed in Section 3.2.

**Scheme 3:**
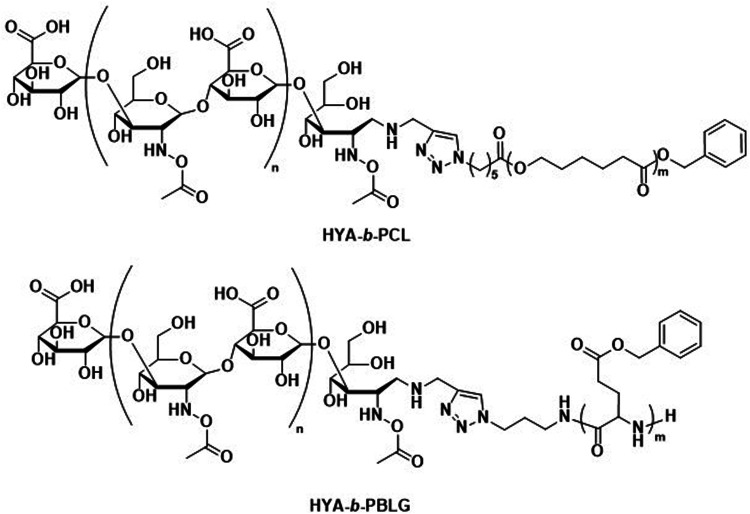
Chemical structures of block copolymers containing hyaluronic acid (HYA) as the hydrophilic block. Combined with biodegradable hydrophobic blocks, their self-assembly leads to fully biodegradable polymersomes. HYA can also bind to CD44, a cell surface adhesion receptor overexpressed in various cancer types, aiding in targeted polymersome uptake (Lecommandoux and coworkers^119^).

The incorporation of naturally available hydrophilic biopolymers in polymersome formulations is another promising alternative to PEG. These building blocks are typically readily available in large volumes and are already extensively used in cosmetic products. As a result, their biocompatibility and degradation mechanisms are well established.^112^ Polysaccharides present a particularly advantageous class of biodegradable and biocompatible polymers with existing approval of both the EMA and FDA.^113–115^ In order to generate amphiphilic block copolymers based on polysaccharides, click chemistry, such as the copper-catalysed azide-alkyne cycloaddition, is often used for their conjugation with other polymers. However, this synthetic approach may be problematic due to the toxicity of residual copper. Schönherr *et al.* showed that (hyaluronic acid)-*b*-PCL (HYA-*b*-PCL) copolymers could form polymersomes (Scheme 3).^116^*In vitro* studies using the enzyme bovine hyaluronidase showed rapid and fast degradation within 30 minutes in acetate buffer (pH 5.6) at 37 °C. Although the study was not extended to *in vivo* tests, hyaluronidases are prevalent in humans^117^ and the presence of hyaluronic acid in human skin, eyes, and joints as well as in cosmetics and biomedical applications makes critical biocompatibility information more readily available.^118^ Lecommandoux *et al.* combined PAmAs with polysaccharides to form the block copolymer HYA-*b*-PBLG (Scheme 3), which was subsequently self-assembled into polymersomes. These structures were rigorously evaluated for the *in vivo* delivery of DOX towards Ehrlich ascites tumour.^119^ Pharmacokinetic parameters showed significant improvement for encapsulated DOX compared to free DOX. A direct comparison to liposomal DOX delivery formulations was, however, not undertaken. Both the DOX-loaded polymersomes and the empty reference polymersomes showed low toxicity, and the reference polymersomes were found to be less toxic than the ones loaded with DOX. The HYA-*b*-PBLG polymersomes also showed a high tumour-to-muscle uptake ratio, owing to the fact that the CD44 receptor present in these tumours readily binds to HYA. Thus, the combination of PAmA and polysaccharides yielded a sophisticated delivery system from simple building blocks that are highly biocompatible and biodegradable.

**Scheme 4:**
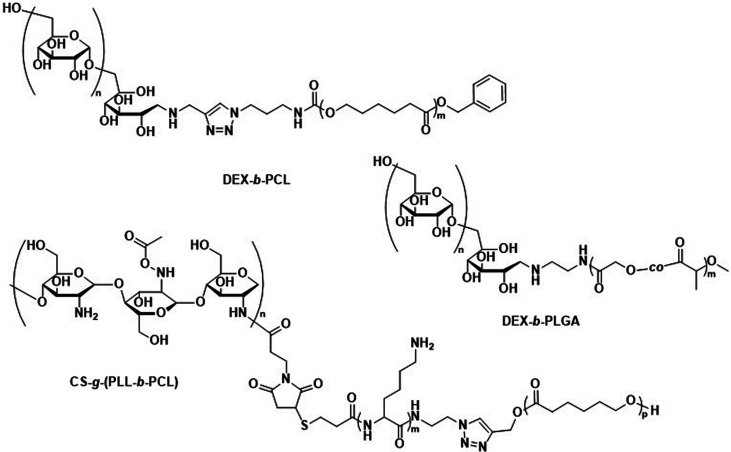
Chemical structures of block copolymers containing dextran (DEX) and chitosan (CS) as the hydrophilic block. Their self-assembly leads to fully biodegradable polymersomes. DEX-*b*-PLGA polymersomes loaded with insulin for oral delivery withstand acidic conditions of the gastrointestinal tract, only degrading under intestinal conditions when pH reaches 7.4 for sustained insulin release (Hadizadeh and coworkers^121^). CS containing polymersomes with an enzymatically cleavable PLL block did not undergo enzymatic degradation of the PLL block due to electrostatic repulsion of the CS with trypsin (Menzel and coworkers^122^).

While there are no studies in which the biodegradation of polysaccharide-based polymersomes was specifically studied, the biodegradation of dextran-*b*-PCL (Scheme 4) nanoparticles by enzymes such as lipases or dextranases has been investigated.^120^ In the presence of dextranases, the degradation rate was found to depend on the weight fraction of dextran, with a degradation of 81% after 48 h for a copolymer containing 33 wt% of dextran. By contrast, the degradation was much faster in the presence of lipases, and extensive degradation was observed after only a few minutes. Preliminary studies on Dex-*b*-PLGA polymersomes (Scheme 4) for oral insulin delivery were conducted by Hadizadeh *et al.*^121^ The reported polymersomes survived the harsh acidic conditions in the gastrointestinal tract, while at pH 7.4 an increased water penetration caused by the dextran in the polymersome membrane led to gradual degradation.^121^ This was manifested in a sustained insulin release under simulated intestinal conditions, while negligible premature release under gastrointestinal tract conditions was detected. An interesting degradation behaviour was also reported for chitosan-based polymersomes by Menzel *et al.*, who assembled chitosan-*g*-[poly(l-lysine)-*b*-PCL] graft copolymers into polymersomes (Scheme 4).^122^ Chitosanase could effectively degrade the β-1,4-linkages of chitosan in the graft copolymer and led to the disassembly of the polymersomes, whereas trypsin was unable to access and degrade the poly(l-lysine) component. This was explained by repulsive electrostatic interactions between the enzyme and the chitosan at physiological pH, reaffirming that the intended cleavable units in the polymersome membrane must be accessible to enzymes. While humans do not produce chitosanase, other enzymes such as lysozyme or chitinases found in the human body are capable of biodegrading chitosan.^123^

Various other polysaccharides have also successfully been used to generate assembled vesicles, such as dextran-*b*-PBLG (Dex-*b*-PBLG),^124,125^ xylan-*b*-(methyl oleate)/(methyl ricinoleate),^126^ and hydroxyethyl starch (HES)-*g*-lauric acid/palmitic acid/stearic acid.^127^ Especially, hydroxyethyl starch, a “semisynthetic”, water-soluble polysaccharide is well known and widely available, with detailed pharmacokinetic and toxicology data available.^128–130^ By varying the degree of hydroxyethylation and the C2/C6 ratio of hydroxyethylation, the rate of metabolic degradation can be controlled.^131^ Mäder *et al.* showed that grafting fatty acids to HES leads to copolymers which could be self-assembled into vesicles.^127^ Unfortunately, neither the degradation kinetics of these vesicles nor their compatibility with cells were reported.^127^ Thus, while HES is interesting as a biodegradable, hydrophilic polysaccharide, its application in polymersomes requires further investigation.

Poly(hydroxyalkanoate)s (PHAs), which are produced by a wide range of microorganisms, represent another class of biopolymers with a property profile that is useful for biomedical applications. These biodegradable polyesters are based on a range of different hydroxyalkanoates that influence their properties and can be naturally degraded by digestive enzymes (amylases, lipases, *etc.*).^132,133^ Langlois *et al.* synthesised PHA-containing block copolymers through thiol-ene additions of PEG to poly(3-hydroxyoctanoate-*co*-3-hydroxyundecenoate) (PHOU), which were assembled into polymersomes.^134^ While in this study neither the pharmacokinetics nor the degradation were explored, it serves as the only example of polymersome formation of PHA-based building blocks, although several publications have reported the inclusion of PHAs in amphiphilic block copolymers.^135,136^

Overall, the use of PAmAs and biopolymers in block copolymers presents a genuine avenue for polymersomal therapeutics, as established biodegradability and biocompatibility data for the polymer blocks are easily accessible. As alternatives to PEG, they alleviate issues surrounding PEG-related immunogenic responses and accelerated clearance upon repeated administrations. While currently few comprehensive *in vivo* studies and comparisons to liposomal species exist, the existing literature suggests strong potential for future investigations regarding their performance as delivery agents. Overall, these types of polymersomes remain underexplored at present.

### 3.2. Enzyme-specific degradable units for controlled polymersome disassembly

As briefly mentioned before, is possible to tailor the biodegradability and cargo release of polymersomes through the inclusion of enzyme-degradable linkers that connect the two blocks of the copolymer. Cleavage of these specific units results in the separation of the different blocks and the loss of amphiphilic character, and thereby leads to the collapse of the polymersomal morphology and release of the cargo. This enables an accelerated degradation of the biodegradable blocks, ensuring a rapid release of encapsulated species, and the renal clearance of the resulting free polymer chains. Many polymersomes have been successfully generated based on this principle and were demonstrated to be cleavable by enzymes that are overexpressed in tumour cells.

**Fig. 4:**
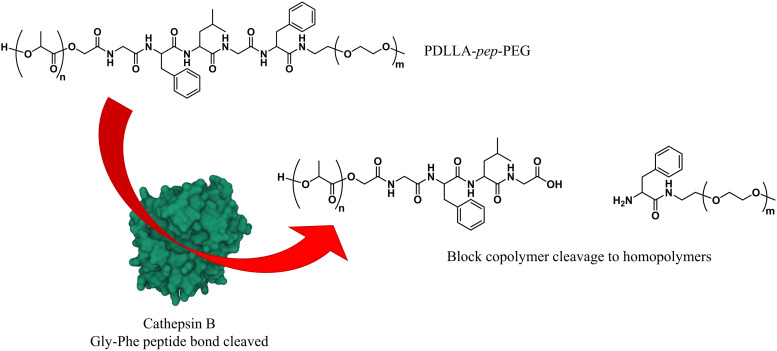
Block copolymers containing the peptide sequence Gly-Phe-Leu-Gly-Phe can be used to form polymersomes that disassemble upon cleavage of the peptide *via* cathepsin B proteolysis (Feijen and coworkers^138^).

Enzyme-specific cleavable linkers for the degradation of polymer materials have been known since the 1980s,^137^ but for polymersome disassembly this was first explored by Feijen *et al.* They introduced the peptide sequence Gly-Phe-Leu-Gly-Phe (pep) between the blocks of a PEG-*b*-PDLLA copolymer (Fig. 4).^138^ Their degradation was investigated in buffers at pH 5.5 and pH 7.4 at 37 °C in the presence of lysosomal enzyme cathepsin B. The lysosomal environment, which features low pH and distinct enzymes, provides an ideal target for directed polymersome degradation.^106,139^ Full PEG-pep-PDLLA polymersome degradation was observed by dynamic light scattering within 2–7 days at pH 5.5, depending on the concentration of enzymes in solution. This process led to the release of 100% of the encapsulated dye in the presence of cathepsin B after 3 days, demonstrating that such a degradation mechanism can be an efficient means to trigger the release of encapsulated cargo. No enzymatic degradation was observed at pH 7.4 or at pH 5.5 in the absence of enzymes within the same time. When surface-functionalised with antibodies, these polymersomes were preferentially taken up by cancerous cells *in vitro*, although *in vivo* studies that corroborate these findings are currently lacking. However, since cathepsin B is overexpressed in certain cancer cells,^106^ the exploitation of enzyme-cleavable block linkers in polymersomes to achieve efficient and specific cargo release should be very effective. Alibolandi *et al.* also employed this concept by using polymersomes composed of PEG-*b*-poly(lactic acid) (PEG-*b*-PLA) block copolymers in which the two blocks were linked by a synthetic peptide with sequence PVGLIG.^76^ The latter was selected because it can be cleaved by matrix metalloproteinase 2 (MM2), another enzyme that is overexpressed in tumour tissue.^140^*In vitro* studies demonstrated a seven-fold increase in anticancer drug (SN38) release in the presence of MM2 for polymersomes bearing the peptide linker. *In vivo* studies corroborated these findings, showing a higher efficacy at reducing tumour growth for peptide-functionalised polymersomes than reference polymersomes lacking the peptide linker. While the enzyme-specific degradation played a role in the release of SN38, the surface functionalisation of the polymersome with specific proteins (AS1411 aptamer) significantly improved the biodistribution towards C26 cell lines at low concentrations in a short time period (6 h). However, after 24 h or at high concentrations of drug/polymersomes the cell viability of cancer cells did not show a significant difference between surface functionalised targeted polymersomes *vs.* non-targeted treatment.

Currently, the scope of enzyme-specific degradable linkers leading to polymersome disassembly is limited. While the full potential of further block copolymers with enzyme-specific degradable linkers remains to be demonstrated, protease-activated prodrugs that function *via* enzyme-cleavable linkers are well documented, since protease overexpression is closely linked to cancer.^141^ These systems may serve as sources of inspiration for the design of future enzyme-sensitive polymersomes.^141^ The sparse attention that enzyme-cleavable linkers in polymersomes have received likely means that the translation of such polymersomes into approved medicines will take longer than other previously discussed polymersomes. Furthermore, the process of including peptide sequences between polymer blocks increases the synthetic efforts and may require further refinement. Nonetheless, this approach broadens the range of polymers that can be applied; since the polymersome degradation is not related to the degradation of either block, also non-biodegradable polymers can be used. Ultimately, the combination of polymersomes containing an enzyme-cleavable linker and surface-modifying bioligands that promote specific cellular uptake, appear to allow the design of polymersomes that provide robust cargo transport to a target destination, where rapid disassembly and cargo release is ensured.

### 3.3. Partially biodegradable polymersomes

**Scheme 5:**
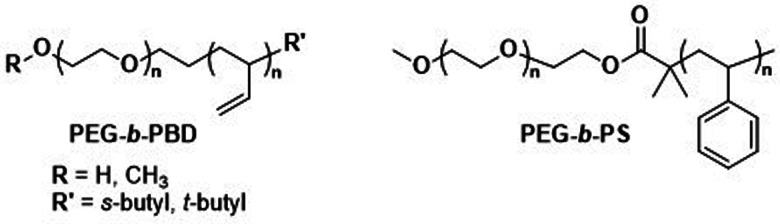
Chemical structures of non-biodegradable copolymers co-assembled with biodegradable copolymers in polymersomes for therapeutic applications.

As mentioned above, the use of a degradable copolymer represents perhaps the most obvious design approach to construct polymersomes that disassemble *in vivo*. However, not the entire polymersome needs to be based on biodegradable polymers to be of interest for drug delivery applications. Indeed, the co-assembly of biodegradable copolymers and non-degradable copolymers can lead to polymersomes whose membranes become porous as the degradation of the biodegradable block copolymer proceeds. At the same time, long circulation times and the overall stability of such polymersomes are retained by the non-degradable block copolymer, providing a potentially worthwhile compromise of properties. Several mixtures of block copolymers have been investigated for this purpose, including PEG-*b*-PCL or PEG-*b*-PLA that were combined with PEG-*b*-poly(butadiene) (PEG-*b*-PBD),^142^ as well as PEG-*b*-PCL mixed with PEG-*b*-PS (Scheme 5).^143^ The cargo release from these systems can be tuned by altering the composition, *i.e.*, the ratio of biodegradable and non-degradable copolymers, which affects the formation rate of pores and their size.^143^ This in turn allows for smaller encapsulants to be released earlier and more rapidly than larger ones. In both instances the poration was shown to eventually lead to a collapse of the polymersome. The pre-clinical evaluation of such copolymer blend polymersomes has, however, not been reported yet, with *in vivo* studies still lacking. Moreover, biocompatibility and pharmacokinetic data remain to be determined.

### 3.4. Summary

In summary, the library of biodegradable and biocompatible polymers is vast, and their self-assembly into polymersomes is by and large well established. The polymer types and block copolymer combinations mentioned here are not meant to be listed; indeed numerous other polymer variations and functional groups have been reported, some of which display properties that render them an attractive basis for use in therapeutic delivery vesicles. In general, simply ensuring that one of the blocks is biodegradable or strategically placing a degradable linker between the blocks is sufficient to enable polymersome degradation and clearance within hours *in vivo*, thereby mitigating the risks associated with accumulation of polymer material inside the body. Moreover, biodegradation should not be overlooked, as by altering the choice of biodegradable block(s) or by incorporating enzyme-selectively cleavable units, cargo release location and rate are affected. By choosing the right polymers, it is possible to endow and modulate the polymersomes’ innate properties. This includes low leakiness during circulation, targeted *in vivo* interactions for cellular uptake and payload release, and rapid excretion following disassembly or degradation. However, the level of detail with which the biocompatibility, degradation and *in vivo* clearance have been addressed in various studies varies greatly. This is perhaps to be expected, as each specific polymersome formulation requires considerable effort to assess if clinical trials are worthwhile, and the extent to which individual research groups wish to pursue this goal will also vary. Nonetheless, the existing literature demonstrates a plethora of polymers may be used to construct polymersome formulations which are biocompatible and biodegradable, and that the biodegradation can furthermore be tuned to occur only within the targeted site for drug release.

## 4. Added functionalities in biodegradable polymersomes

Polymersomes are subject to various dynamic environments during administration, *i.e.*, in the blood-stream, through organ distribution, cellular uptake, and following endosomal escape. Therefore, stimuli-sensitive polymersomes have gained significant interest, as these particles enable spatiotemporal control over disassembly, degradation, and permeability. Functional groups that alter the physical or chemical properties of the polymers in response to environmental conditions have been widely used to create multitudes of stimuli responsive polymersomes.^8,13,144^ These stimuli include various fluctuations that are common to physiological environments such as a change in pH, redox potential, and local enzyme concentrations. In addition, externally applied stimuli, such as light, magnetic fields, and heat may be utilised. Polymersomes that disassemble and/or undergo backbone bond cleavage after the application of such stimuli offer spatiotemporally controlled release as well as accelerated biodegradation and hepatic or renal clearance of the disassembled or degraded polymers. The strategic incorporation of stimuli-responsive units in either block or between blocks allows programmable collapse of the polymersome structure upon exposure to the targeted stimulus, as backbone bonds are cleaved or a switch of the solvent compatibility occurs.^145^ The resulting ‘free’ polymer chains possess significantly smaller hydrodynamic radii, allowing for renal filtration.^146^ Extensive research concerning the development of such stimuli-responsive polymersomes has been undertaken and previous reviews neatly summarise this vast field.^6,8,9,52,147,148^ Considering that many diseases present distinct physiological fluctuations (often also localised in organs or tissues), polymersomes that are capable of responding to pH gradients,^149^ enzyme overexpression,^150^ changing redox potential,^151^ and biochemical reactions^151,152^ are of great interest in the context of drug delivery.

### 4.1. Electrostatic interactions of polymers

Unmodified, biocompatible polymers capable of electrostatic interactions, have been used to improve the drug and protein loading efficiencies, and enhance the endosomal escape of polymersomes. For instance, Eisenberg *et al.* created polymersomes that are capable of adsorbing functional biomolecules onto their surface by exploiting polyelectrolyte-mediated protein adsorption.^153^ Using ABC triblock copolymers consisting of PEG, PCL, and poly(acrylic acid) (PAA; PEG-*b*-PCL-*b*-PAA), all individually FDA approved polymers, polymersomes with an asymmetric membrane were obtained.^153^ The longer, charged PAA segments were preferentially located on the outer surface, enabling electrostatic binding of bovine serum albumin (BSA). By simply changing the length of the various polymer blocks, the same system yielded polymersomes in which the PAA blocks faced the interior and this increased the loading efficiency of the polar drug DOX·HCl, also on account of attractive electrostatic interactions. The buffering capacity of the PAA blocks at endosomal pH ranges further aids in endosomal escape through the “proton sponge” effect (see Section 6.3). These studies on PEG-*b*-PCL-*b*-PAA polymersomes demonstrate that relatively simple block copolymers can afford therapeutically relevant polymersomes that display low toxicity, suitable biodegradability, and versatile applications for ligand functionalisation or improved cellular uptake.^154^ Despite proven degradation and low toxicity both *in vitro* and *in vivo*, further studies to elucidate pharmacokinetics and biodistribution are currently lacking.

Other biodegradable asymmetric triblock copolymers capable of electrostatic interactions were employed by Zhong *et al.*, who used PEG-*b*-PCL-*b*-poly(2-(diethylamino) ethyl methacrylate) (PEG-*b*-PCL-*b*-PDEA) to create polymersomes for high efficiency protein encapsulation.^155^ Like the PAA blocks of the PEG-*b*-PCL-*b*-PAA polymersomes discussed above, the short PDEA blocks were preferentially located on the inside of the polymersomes, allowing for high protein loading through electrostatic/hydrogen bonding interactions of the PDEA blocks during polymersome assembly. Empty polymersomes were found to be non-toxic towards HeLa and RAW 264.7 cells in concentrations of up to 0.5 mg mL^−1^.

### 4.2. Stimuli-responsive polymersomes

#### 4.2.1. Polymersome disassembly triggered by biological stimuli

Within the human body, and between healthy and cancerous cell types, internal biological fluctuations exist and these can be harnessed by using appropriate stimuli-sensitive polymers. For example, variations of the pH are widespread throughout the human body, within subcellular compartments, and the acidic microenvironments of tumours.^156^ The increase in reductive potential upon entering the cells can be exploited for polymersomes with cell-selective cargo release by including redox sensitive blocks.^157,158^ Excess reactive oxygen species (ROS) have also been linked to various diseases.^159^ In cancer cells, increased ROS play a role in their proliferation, which is balanced by increased antioxidant levels to maintain ROS homeostasis for the cancer cells.^160,161^

Zhong *et al.* used a combination of the previously mentioned PEG-*b*-PCL-*b*-PDEA block copolymer alongside PEG-*b*-PCL blocks connected by a disulfide linker (PEG-SS-PCL) to form reduction-sensitive polymersomes.^162^ The polymersomes alone displayed low cytotoxicity and higher weight percentages of PEG-SS-PCL led to enhanced reduction-triggered release of the protein cytochrome *C in vitro*. Further surface functionalisation with β-d-galactose then led to a selective uptake into hepatoma cells (HepG2), where the reduction sensitive release of cytochrome *C* triggered cell death. Since these polymersomes used the previously mentioned PEG-*b*-PCL-*b*-PDEA copolymers, high protein loading efficiencies were maintained while retaining biodegradability.

**Scheme 6:**
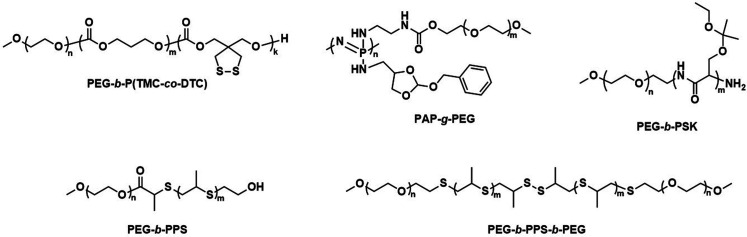
Chemical structures of stimuli-responsive block copolymers used to create polymersomes for stimuli-responsive disassembly. Polymersomes containing PDTC form disulfide crosslinks which break in reductive environments, *e.g.* tumour cytoplasm (Zhong and coworkers^163^). PAP-*g*-PEG and PEG-*b*-PSK polymersomes are pH responsive as ester hydrolysis at pH 5.5 causes a hydrophobic-to-hydrophilic shift and polymersome collapse (Qiu and coworkers,^168^ Gu and coworkers^169^).

Further pre-clinical studies concerning pharmacokinetics, biodistribution, and toxicity of polymersomes and micelles containing the biodegradable and reduction-sensitive polymer poly(trimethylene carbonate-*co*-dithiolane trimethylene carbonate) (P(TMC-*co*-DTC) as the hydrophobic block combined with PEG as the hydrophilic block were performed by the Zhong group (Scheme 6). P(TMC-*co*-DTC) is a copolymer containing the already mentioned biocompatible and biodegradable PTMC backbone, along with dithiolane-functionalised analogues, which facilitate the formation of reduction-sensitive disulfide-cross-links.^163^ These disulfide cross-links are generally stable in extracellular environments, and only de-cross-link once exposed to reductive environments such as the cytoplasm in tumour cells.^164^ These cross-links provide retention of cargo species inside the polymersome during circulation, with release exclusively occurring inside cells upon the cleavage of the cross-linking bonds. Polymersomes based on P(TMC-*co*-DTC) surface-functionalised with targeting ligands were shown to have favourable plasma circulation times in comparison to their free payload. In addition, they were preferentially taken up by various tumours (including blood brain barrier crossing for glioblastoma treatment), exhibited a low inherent toxicity, and enabled an efficient reduction-triggered release of their payload once internalised inside cells.^80,165,166^ As an example, polymersomes formed from PEG-*b*-P(TMC-*co*-DTC) with surface functionalisation of peptides were shown to be effective targeted delivery systems for nucleus accumbens-associated protein-1 (NAC-1) siRNA for the sensitisation of antiangiogenic therapy against metastatic triple-negative breast cancer (TNBC).^167^*In vitro*, these polymersomes showed high colloidal stability until exposed to 10 mM glutathione (GSH). Significant destabilisation after 4 hours was evident *via* DLS, confirming that siRNA release could be achieved in reductive intracellular environments.^167^*In vivo*, these polymersomes remained stable with long plasma half-lives of *t*_1/2_ = 9.3 h. When siRNA-loaded polymersomes were combined with bevacizumab, an antiangiogenic agent used in breast cancer treatment, effective TNBC suppression was observed. The mice's body weight remained stable throughout the combined treatment indicating negligible toxicity. In addition, the survival time of mice treated with both polymersome and bevacizumab was significantly extended.

The potential of P(TMC-*co*-DTC) based polymersomes for targeted intracellular drug delivery was further highlighted with direct comparisons to liposomal formulations.^79,151^ PEG-*b*-P(TMC-*co*-DTC) polymersomes, surface-functionalised with a cyclic peptide (cNGQGEQc) for targeted DOX delivery to human lung cancer cells, showed an improvement of therapeutic properties over Lipo-Dox®. *In vivo*, the polymersomes showed excellent biocompatibility; with Dox-loaded polymersomes showing a maximum-tolerated dose at least 6-fold higher than Lipo-Dox®, along with substantially improved biodistribution towards tumour cells. This study exemplifies how stimuli-responsive units in polymersomes can facilitate efficient on-site release, synergising well with the otherwise low-leakage of polymersomes. This reduces the harmful side effects which plagues the leakier membrane of liposome formulations as observed for Lipo-Dox®, which showed significant hepatotoxicity and nephrotoxicity in mice compared to minimal toxicity of Dox-loaded polymersomes based on PEG-*b*-P(TMC-*co*-DTC).^79,170^

Qiu *et al.* recently demonstrated the effectiveness of pH-responsive polymersomes as delivery agents for DOX.^168^ Polymersomes assembled from poly(phosphazene)-*graft*-PEG (PAP-*g*-PEG) copolymers (Scheme 6) rapidly collapsed at pH 5.5, because of the hydrolysis of the *ortho* ester side groups on the PAP backbone. This mechanism allowed for an accelerated release of DOX–HCl, rendering the drug release sensitive to tumour environments. An interesting feature of these polymersomes is their high drug loading capacity, which can be attributed to the fact that the *ortho* ester 4-aminomethyl-2-benzyloxy-[1,3]-dioxolan interacts with DOX·HCl through hydrogen bonding and π-stacking. The polymersomes themselves were non-toxic at concentrations of up to 5 mg mL^−1^ towards mouse sarcoma cells. *In vivo* studies (body weight, histopathology) in mice further supported that DOX·HCl-loaded polymersomes display a low toxicity towards normal tissues. *Ex vivo* fluorescence studies confirmed a high presence of DOX·HCl within tumours 24 h post-injection. One drawback was that even when 20 wt% cholesteryl hemisuccinate was incorporated into the polymersomes to prevent premature drug leakage, 28% of DOX·HCl was released at 7.5 pH over 24 h.

Polymersomes that are pH-responsive were also created using cleavable units such as cyclic benzylidene acetals,^171^ imine-DOX prodrugs,^172^ and hydrazone linkers.^173^ A low cytotoxicity could be maintained when these functional units were introduced into biocompatible polymers, making them attractive candidates for controlled release systems.^174,175^ Polymersomal collapse can occur when the cleavage of the pH-sensitive units results in hydrophilic groups within the initially hydrophobic polymer block segment and may be followed by DLS, transmission electron microscopy (TEM) and scanning electron microscopy (SEM).^173,175,176^ Polymer blocks with ionisable side-chains have also been successfully used to impart pH-responsiveness and subsequently enable release of proteins and polymersome disassembly.^176–178^

Glucose-mediated interactions can also act as an *in vivo* stimulus for polymersome disintegration.^179^ Glucose oxidase (GOx) encapsulating poly(ethylene glycol)-*block*-poly(propylene sulfide)-*block*-poly(ethylene glycol) (PEG-*b*-PPS-*b*-PEG) polymersomes disassembled when glucose entered the interior and caused GOx to produce H_2_O_2_, which subsequently oxidised the PPS block and rendered it hydrophilic.^179^ Ketal-modified poly(serine) (PSK) and PEG block copolymers (PEG-*b*-PSK, Scheme 6) were further exploited to assemble glucose-responsive, pH-sensitive polymersomes.^169^ When glucose diffused into these polymersome's interior, encapsulated GOx mediated the conversion of glucose to gluconic acid, which caused a pH drop within the polymersome. This then led to the hydrolysis of the acid-labile ketals, yielding a fully hydrophilic block copolymer and polymersome disassembly. Sugar-responsive polymersomes may also be generated by incorporating boroxole polymer blocks, which can bind to pyranosides such as glucose and as a consequence transition from hydrophobic to hydrophilic.^152^

Enzyme-mediated disassembly (not biodegradation) has been achieved using polymersomes composed of amphiphilic copolypeptides, where hydrophilic oxidised methionine residues can be reduced by reductases, yielding hydrophobic methionine.^180^ Polymersomes comprised of poly(l-methionine)-*block*-poly(l-leucine-*stat*-l-phenylalanine) copolypeptides with oxidised methionine blocks underwent a conformational change (linear to α-helical) and water-solubility change when exposed to reductase enzymes.

#### 4.2.2. Polymersomes repsonsive to external stimuli

Concurrent to polymersomes designed to respond to internal biological stimuli, polymersomes designed to enhance permeability/disassemble upon application of external stimuli have also been realised. Numerous stimuli have been explored, although in the context of polymersomes that serve as vectors for drug molecules, proteins and genes, temperature and light have received most of the attention, due to the simplicity with which these stimuli can be applied. Furthermore, treatments against cancer cells have shown improved efficacy when combined with localised heating, *e.g.* through hyperthermia or photothermal therapy.^181–183^

This approach is exemplified by a pre-clinical study by He *et al.* on biocompatible polymersomes in which a spatiotemporal controlled disassembly was achieved through synergistic interactions of photosensitizers with the oxidation of stimuli-responsive blocks.^184^ The authors studied PEG-*b*-PPS polymersomes (Scheme 6) that contained the photosensitizer zinc phthalocyanine (ZnPc) in the hydrophobic shell and were loaded with DOX·HCl in the aqueous lumen. ZnPc had previously been applied in photodynamic therapy (PDT), a treatment method that has received FDA approval.^185^ The possibility to utilize these polymersomes as near-infrared light (NIR) triggered, self-immolative drug carriers for cancer therapy was explored.^184^ Irradiation with 660 nm light enabled ZnPc to oxidise the sulphur atoms of the PPS block. The resulting hydrophobic to hydrophilic transition led to the disassembly of the polymersome and drug release. These co-loaded polymersomes exhibited a low cytotoxicity in the absence of NIR light. Upon irradiation with NIR light, however, the system displayed effective cytotoxicity towards malignant melanoma *in vitro* and in *in vivo* studies involving nude mice.^184^ Biodistribution studies of these polymersomes showed predominant accumulation in the tumour, likely due to the enhanced permeability and retention (EPR) effect,^186^ and in the liver. Since the area of illumination can be controlled, the selective drug release inside malignant melanoma is possible. Polymersomes present a distinct advantage over liposomes in this context, since their thick hydrophobic membrane enables the co-loading of ZnPc with DOX·HCl which overcomes the problem of the insolubility of the photosensitizer and aggregation issues in water. Loading of ZnPc and DOX·HCl into PEG-*b*-PPS polymersomes thus provides a synergistic photodynamic therapy and chemotherapeutic approach, where side effects are reduced by selective irradiation of the tumour.

Other light-responsive polymersomes were made by attaching photochromic molecules as side chains in the block copolymers, including azobenzenes, which display light-induced *trans–cis* isomerisation, leading to conformational changes or polymersome bursting.^145,187^ Further, spiropyrans and donor-acceptor Stenhouse adducts (DASAs) have been employed to reversibly control the membrane permeability of polymersomes upon irradiation.^188,189^ The introduction of photocleavable linkers between blocks present another means of on-demand polymersome collapse; a frequently utilised motif for this purpose is the *ortho*-nitrobenzyl unit.^190,191^

**Scheme 7:**
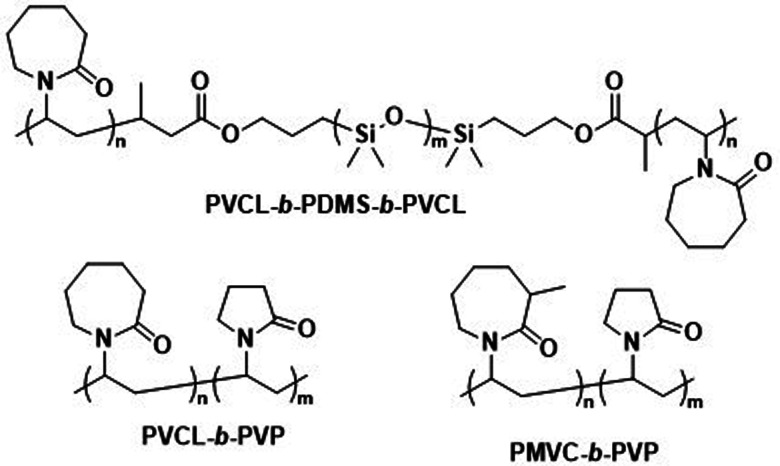
Chemical structures of temperature-responsive block copolymers used to create polymersomes for temperature-sensitive disassembly or release. PVCL and PMVC have a LCST and, in combination with hydrophobic PDMS or the hydrophilic PVP blocks, form polymersomes (Kharlampieva and coworkers^192–194^).

Kharlampieva and coworkers performed several studies on temperature-responsive polymersomes for controlled drug delivery.^192,193^ Poly(*N*-vinylcaprolactam)-*block*-PDMS-*block*-poly(*N*-vinylcaprolactam) (PVCL-*b*-PDMS-*b*-PVCL, Scheme 7) polymersomes were found to increase the release of DOX·HCl when heated above their lower critical solution temperature (LCST), which, depending on the PVCL block length, was between 37 °C and 42 °C. Apart from their temperature-responsiveness, PVCL blocks are biodegradable and biocompatible, making them ideal choices for biomedical applications. Above the LCST, the H-bonding of water with PVCL is disrupted, causing the collapse of the PVCL chains. This leads to shrinkage of the polymersomes and causes drug release. Furthermore, it was shown that at pH 3 the ester linkages between PVCL and PDMS blocks can be cleaved, which suggests that the degradation of the entire polymersome within endosomal/lysosomal compartments may be possible. This would allow for complete drug release and rapid renal clearance of the polymer. By replacing the hydrophobic PDMS with a hydrophilic block, an inverse approach using thermo-responsive polymersomes was achieved with PVCL-*block*-poly(*N*-vinylpyrrolidone) (PVCL-*b*-PVP; Scheme 7). The polymersome self-assembly was carried out at elevated temperatures (48 °C, *i.e.* above the LCST of the copolymer) and the structure was subsequently “locked” by adding tannic acid, which stabilised the morphology through H-bonding.^193^ The biocompatibility of these polymersomes was confirmed at concentrations of up to 100 μg mL^−1^. Upon degradation of the tannic acid stabiliser *via* intracellular enzymes, the collapse of these polymersomes and drug release ensued, since the physiological temperature is below the LCST of the copolymer. A further variant of these polymersomes, in which poly(3-methyl-*N*-vinylcaprolactam) (PMVC; Scheme 7) was employed instead of PVCL, allowed lowering the LCST to below 20 °C, so that stable polymersomes formed at room temperature.^194^ DOX·HCl-loaded PMVC-*b*-PVP polymersomes showed excellent biocompatibility and in contrast to DOX·HCl-loaded liposomes did not cause damage to the heart, lungs or spleen. However, cargo release from PMVC-*b*-PVP polymersomes has yet to be demonstrated, and will likely require additional functionalisation of the copolymer.

In summary, stimuli-responsiveness can be imparted to polymersomes, while biodegradability and biocompatibility are retained. In some cases, the stimuli-responsiveness was provided by the biodegradable block. The benefits of stimuli-responsive permeability modulation or total disassembly of polymersomes, directed towards intracellular environments, in combination with surface-functionalised targeting ligands that cause preferred uptake by malignant cells are clearly highly attractive features. Oftentimes the block copolymers forming the stimuli-responsive polymersomes are complex and novel, and this raises questions about their scalability and potential approval by medicinal agencies, likely delaying the translation into the clinic. Furthermore, comparisons to liposomal species remain necessary as stimuli-responsive liposomes species are also known in literature.^195–197^

Modulating the polymersomes’ propensity to release their cargo and/or to disassemble upon application of an external stimulus has also been achieved with other external stimuli such as magnetic fields, electric fields, ultrasound, mechanical force, or enzymatic cleavage.^13,145,198,199^ These triggers have been less extensively explored, yet the available studies highlight the broad range of possibilities that are available to tune the release properties of polymersomes, enable their disassembly, and ensure efficient clearance of the building blocks from the body.

### 4.3. Polymersomes with lipid-like block copolymers

Polymersomes that contain blocks with lipid-like side chains, such as phosphorylcholines and cholesteryl motifs, have been successfully used as biomedical delivery vehicles. These polymersomes combine the highly biocompatible nature of lipids with the design opportunities offered by the vast library of possible polymer combinations.

**Scheme 8:**
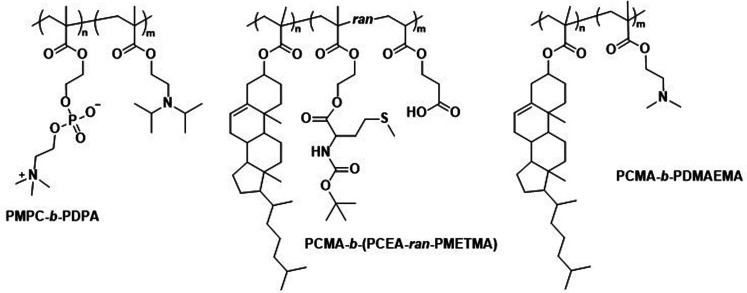
Chemical structures of copolymers combining lipid species and synthetic polymers for lipid-like and hybrid polymersomes. PMPC is a biomimetic, nonfouling, and nonantigenic polymer, while the PDPA block endows polymersomes with pH-responsive collapse. The resulting polymersomes were used for DNA encapsulation (Battaglia and coworkers^200^). PCMA based copolymers co-assembled with lipids yield hybrid vesicles, thereby combining the synthetic engineering of polymers and with the facile self-assembly of lipids (Städler and coworkers^203,204^). Furthermore, PCMA is biocompatible, biodegradable and aids in forming well-defined nanostructures when contained in amphiphilic block copolymers.

Poly(2-(methacryloyloxy)ethyl-phosphorylcholine)-*block*-poly(2-(diisopropylamino)ethyl methacrylate) (PMPC-*b*-PDPA; Scheme 8) diblock copolymers were used by Battaglia *et al.* to produce polymersomes loaded with DNA, antibodies, and cytokines.^200^ These polymersomes exhibit a pH-dependent collapse upon protonating the tertiary amine groups in the PDPA block at pH 5–6, resulting in the release of the encapsulated DNA. This pH-dependent dissociation makes these polymersomes attractive for intracellular delivery through endosomes, where pH values drop from 7.4 to the required range of 5–6. In addition, the authors proposed that the sudden increase in the concentration following polymersome dissociation leads to osmolysis of endosome membranes, allowing efficient cargo delivery into the cytosol. The cytotoxicity of these polymersomes was low, with near to no effect on human dermal fibroblast cells (HDF). Cytotoxicity was only observed in exposure studies involving Chinese hamster ovary (CHO) cells at a concentration of 2 mg mL^−1^. The system was compared to the commercial liposome transfection agent Lipofectamine TD by delivering green fluorescent protein (GFP)-encoded DNA plasmid into HDF and CHO cells. PMPC-*b*-PDPA polymersomes showed a higher transfection efficiency and cell viability for both cell types than Lipofectamine TD. By outperforming commercial liposomes *in vitro*, these polymersomes highlight the beneficial aspect of “borrowing” lipid biocompatibility and combing this treat with the stimuli-responsive characteristics of a synthetic polymer for directed DNA delivery. PMPC-*b*-PDPA-based polymersomes have further shown potential for the targeted delivery of drugs towards glioma in *in vitro* studies.^201^ In these studies, PMPC-*b*-PDPA polymersomes were loaded with antimycobacterial drugs to target intracellular pathogens in macrophages. The vesicles showed good biocompatibility at a concentration of 1 mg mL^−1^.^202^*In vivo* studies of these polymersomes conjugated with Cy5 dye in zebrafish showed rapid accumulation in target cells 10 minutes post-injection, with a general preference for uptake by macrophages.

### 4.4. Hybrid polymer–lipid vesicles

In addition to the use of polymers with pendant lipid functions, the combination of amphiphilic lipids and polymers is another design option for polymersomes. Städler *et al.* co-assembled vesicles from polymers and lipids, and demonstrated the possibility to harness the properties of both amphiphilic block copolymers and lipids within these ‘hybrid’ vesicles. The block copolymers used were a combination of poly(cholesteryl methacrylate) (PCMA) with a statistical copolymer of methionine methacryloyloxyethyl ester (METMA) and 2-carboxyethyl acrylate (CEA; Scheme 8).^203^ METMA was chosen because it can be readily functionalised, and CEA was used to promote lysosomal escape. A mixture of the block copolymer PCMA-*b*-p(CEA-METMA), Lissamine-rhodamine B-modified 1,2-dimyristoyl-*sn-glycero*-3-phosphoethanolamine lipids (L^rho^), and 1,2-dioleoyl-*sn-glycero*-3-phosphocholine (DOPC), which is already used in approved liposomal formulations, was assembled into hybrid-vesicles *via* film rehydration. The presence of both polymers and lipids was confirmed by two distinct emission peaks in the fluorescence spectra that originated from fluorescence resonance energy transfer (FRET) reporter pairs attached to both the lipid and polymer. Measurements of the mean cell fluorescence after uptake of these hybrid vesicles by HepG2 cells over 6 h followed by 24 h incubation indicated that the lipids were either being degraded or exocytosed, while the polymers were retained within the cells.

Further work involved hybrid vesicles formed from mixtures of PCMA-*b*-poly(2-(dimethylamino)ethyl methacrylate) (PDMAEMA; Scheme 8) block copolymers, phospholipids such as POPC, and rhodamine-labelled phosphoethanolamines.^204^ These vesicles were designed to support host defence in macrophages against pathogens such as *Mycobacterium tuberculosis* by loading cells with enzymes for intracellular NO production.^204^ Notably, the PDMAEMA block, used to aid in endosomal escape *via* the “proton sponge” effect, was linked to toxicity. However, cell viability of RAW 264.7 cells could be maintained by sequential incubation with smaller doses of hybrid vesicles rather than one large dose, while retaining control over cell internalisation. Hybrid vesicles loaded with β-galactosidase could convert β-galactosyl-pyrrolidinyl diazeniumdiolate into NO inside RAW 264.7 cells. At a vesicle concentration of 0.02 mg mL^−1^, β-galactosidase-loaded hybrid vesicles were inherently non-toxic and produced NO in non-toxic quantity (assessed by cellular dehydrogenase activity).

By combining lipids with polymers, lipid biocompatibility can alleviate certain toxicity concerns that are linked to the use of cationic polymers to exploit the “proton sponge” effect for lysosomal/endosomal escape. Conversely, the introduction of amphiphilic block copolymers into lipid systems could address issues of leakiness and functional limitations of liposomes.

### 4.5. Summary

Employing only biodegradable and biocompatible block copolymers, it remains feasible to exploit a vast library of copolymers to generate polymersomes for site-selective delivery of drugs, proteins, and genes. A high loading efficiency and rapid, spatiotemporally controlled release of the cargo has also been made possible by including stimuli-responsive motifs in the copolymers. In summary, biodegradability and biocompatibility do not prevent efficient and effective therapeutical polymersome design. In addition, the ability to tailor the degradation and disassembly through functionalisations and choice of polymer allow polymersomes to distinguish themselves with respect to other drug delivery methods, especially liposomes.

## 5. Scalable, efficient, and reproducible polymersome preparation

The “self-assembly” of amphiphilic block copolymers into polymersomes is generally an active process (hence, the term assembly may be more appropriate) that affects or determines the shape, size, size distribution, and loading efficiency of the nanostructures. More than ten different techniques are frequently used (Table 1) to self-assemble amphiphilic block copolymers into polymersomes. These methods vary considerably in complexity, required equipment, and throughput.^4,15^ Many of these approaches were originally developed several decades ago to create liposomes and have been adopted to create polymersomes. Extensive discussions of these techniques are provided in previous articles, and the reader is directed to them for detailed information.^18–20,53,205^ Below, the two most commonly used techniques are outlined before several, more advanced techniques are discussed.

**Table 1:** Overview of self-assembly techniques for polymersome and liposome production

Technique	Equipment required	Continuous manufacturing amenability	Polymer or lipid concentration (% w/v)	Typical throughput	Polymersome diameter (nm)	PDI	Loading efficiency (%)	Comments	Ref.
Polymersomes									
Solvent exchange	Basic lab equipment	Not amenable	<1	1–5 mL per batch	<100	—	<1–25	No PDI values prior to extrusion or SEC available	4,189,206,207
Thin film rehydration	Basic lab equipment	Not amenable	<1	1–5 mL per batch	<100 (up to 5000 nm reported)	0.28–0.56	5–15	PDI values before extrusion or SEC	4,15,208
Flash nanoprecipitation	Specialised equipment	Minor adaptations required	Before mixing 4–10	1 to 8 kg day^−1^	65–300	0.18–0.30 (up to 0.63 reported)	16–43	Throughput mentioned, not tested	209–212
			After mixing 1.6–4						
Stirred tank reactors	Specialised equipment	Highly amenable	Before mixing 20	0.012–1.5 L	30–180	0.17–0.19	Not reported		213
			After mixing 1						
Microfluidics	Specialised equipment (including commercially available systems)	Amenable	<5	In the order of μl min^−1^	40–1 000 000	0.05–0.15	29–84	Throughput up to mL min^−1^	214–219
PISA	Basic lab equipment	Adaptations required (*i.e.* flow reactor)	10–50 wt%	Usually <20 mL per batch	230–760	0.04–0.20	9–79	In flow reactor up to 60 g polymer structure per day	220–224
Liposomes									
Non-double emulsion microfluidics	Commercial systems available	Highly amenable	<5	<1 mL up to 20 L h^−1^	50–800	0.05–0.15	20–>99		225–228
Octanol-assisted microfluidic double emulsion	Specialised equipment (lithography)	Highly amenable	0.1–0.5	25–75 liposomes per second	5000–20 000	—	Very high	No value reported for PDI	229,230
Supercritical fluids	Highly specialised equipment and expertise	Highly amenable	0.5–1.5	Not reported	150–300	0.17–0.20	58–96		231

### 5.1. Conventional methods and workflow to prepare polymersomes

Polymersomes can be produced by various methods that can be classified into solvent-based and solvent-free methods. Solvent exchange can be used to induce a nanoprecipitation process in which the solvent is switched from a water-miscible, fully solvating solvent of the block copolymer, such as THF, to one that only solvates one of the blocks, typically water. This is usually carried out by dripping a solution of polymer in a water-miscible organic solvent into an aqueous phase under vigorous stirring, by adding water to the polymer solution dropwise under stirring, or by dialysing the polymer solution against a large volume of water or buffer. The process is fast, requiring typically only a few minutes, does not need special equipment, and can be carried out with limited technical expertise.

Film rehydration is a popular solvent-free method, in which a fully solvated block copolymer solution, *e.g.* in THF, is dried to form a thin film. Block copolymers that are suitable to form vesicles will form a lamellar phase in the bulk of these films. When exposed to water, it will enter into the thin film through small defects, the lamellae will bulge and peel off from the surface and form vesicles. Both techniques are swift and robust processes that can be easily implemented in the lab, which is the reason for their ubiquitous use. However, a variety of structures and aggregates typically form in addition to the desired vesicles so that extrusion and purification steps are required – see below. For more details on these classical methods to prepare polymersomes, we refer the reader to some excellent reviews that cover the common solvent-based and solvent-free methods.^4,15,16^

In both of the above-mentioned techniques, loading of the polymersomes can be achieved by adding a cargo to the aqueous or organic phase during self-assembly. This cargo can be a protein, a drug, or dye molecule that is hydrophilic or hydrophobic. If the cargo is hydrophilic, it is encapsulated in the aqueous lumen of the polymersome, while hydrophobic payloads accumulate in the hydrophobic membrane of the polymersome. The encapsulated amount of a payload *versus* the total amount of it employed during vesicle formation is referred to as encapsulation efficiency. It depends on a variety of factors, such as the preparation method of the vesicles, the concentration of the payload, the total volumes of solvent used (*i.e.* dilution), whether any interactions between the encapsulant and the polymer lead to an active association of the cargo with the polymersome, or if the intermediate structures that form during self-assembly can already host the cargo or not.

For the encapsulation of hydrophilic cargo such as proteins or hydrophilic dyes, and assuming that the concentration of cargo inside the formed vesicles is equal to the concentration of the cargo in the continuous phase before self-assembly, the overall encapsulation efficiency is limited by the volume fraction of the vesicles, more precisely by the volume fraction of the solution inside the vesicles.^232^ Based on geometric considerations and the fact that vesicles form in dilute aqueous conditions, the highest attainable volume fraction was estimated to be below 10%.^232,233^

The loading of proteins into polymersomes is most often carried out by the film rehydration method to avoid exposure of the proteins to organic solvents, and commonly results in loading efficiencies between 5–15%.^15^ For example, film rehydration resulted in an encapsulation efficiency of 5% for hemoglobin and BSA in polyethyleneoxide-*block*-polyethylethylene polymersomes,^234^ and between 3 and 12% for hemoglobin in PEG-*b*-PBD polymersomes.^235^ The encapsulation efficiency of laccase in poly(*N*-vinylpyrrolidone)-*block*-polydimethylsiloxane-*block*-poly(*N*-vinylpyrrolidone) polymersomes was reported to be 10–20% after film rehydration,^236^ and the encapsulation efficiency of ovalbumin in PEG-*b*-PPS polymersomes was 9%.^237^

Given the high costs of therapeutic proteins, a high encapsulation efficiency is desirable for an economic and scalable preparation of polymersomes-based drugs. Various methods have been developed to increase the loading of proteins and enzymes into polymersomes, such as electroporation^238^ or a direct hydration method.^237^ In the latter, a water soluble PEG homopolymer was mixed with the amphiphilic PEG-*b*-PPS block copolymer. Self-assembly was then carried out by placing the polymer mix into an ovalbumin containing aqueous solution, resulting in 37% encapsulation efficiency, probably because the proteins got entrapped into an intermediate sponge phase that then dispersed into polymersomes.^237^ This method was further refined into a “progressive saturation” method in which an aqueous protein solution is added in several aliquots to a mixture of PEG and an amphiphilic block copolymer, assisted by sonication in each step, which greatly enhanced the encapsulation efficiency of various proteins in PEG-*b*-PBD polymersomes, *e.g.* up to 56% for immunoglobulin G.^239^ However, the encapsulation efficiency of some proteins such as haemoglobin and myoglobin remained below 10%.^239^ Thus, the type of protein influences the incorporation into polymersomes as much as the encapsulation method, probably because an accumulation of the protein in the self-assembled structures requires an attractive interaction between the protein and the polymer, such as hydrophobic interactions or electrostatic interactions. Another possibility to increase the encapsulation of proteins into polymersomes is the extrusion step commonly applied for size homogenisation (see below), in which vesicles are deformed and temporarily broken up when being squeezed through small pores. For example, a hollow fibre extrusion increased the encapsulation of proteins into polymersomes by a factor of two compared to conventional extrusion through track etched membranes.^240^

It should be noted that the mechanism of vesicle formation, which depends on the block copolymer and the self-assembly method,^15,241^ can greatly influence the encapsulation efficiency. If the inner aqueous pool of the polymersomes is not readily accessible for the encapsulants, the concentration of the cargo inside of the final polymersomes will be significantly reduced compared to the loading solution. During film rehydration, the evolution of structures on the surface of the film can proceed from the lamellar phase to an interconnected sponge phase, to hexagonally packed vesicles, and finally to the dispersion of polymersomes.^241^ In essence, the lamellae that bulges off the polymer film will curve towards the film, thereby excluding the potential cargo from being encapsulated into the polymersome,^239^ unless it binds into the lamella phase or the sponge phase. Thus, the encapsulation of myoglobin into PEO-*b*-PBD polymersomes by thin film rehydration was found to be very inefficient.^239^

Another example of low encapsulation efficiency was reported by Adams *et al.* for the pH-switch induced self-assembly of polyethyleneoxide-*block*-poly(*N*,*N*-diethylaminoethyl methacrylate) (PEO-*b*-PDEAMA) polymersomes.^233^ The encapsulation efficiencies of the hydrophilic dyes Rhodamine B and riboflavin were significantly lower than expected from the concentration of the dyes in solution. Most likely, self-assembly proceeded through structures without an inner solvent pool such as micelles, that then rearranged to form an internal compartment that grew in volume by diffusion of water into the self-assemblies, excluding any cargo that could not permeate through the hydrophobic parts of the self-assemblies.^233^ If, however, there are non-covalent attractive interactions between the cargo and the self-assembling polymers, such as electrostatic interactions between the payload and oppositely charged polymer blocks, a much higher loading efficiency can be achieved. For example, 20% loading efficiency in film rehydration was reported for the encapsulation of (negatively charged) DNA into polymersomes that comprised a protonated cationic polymer block in acidic conditions,^200,242^ and the loading efficiency could be further increased up to 55% by application of ultrasound.^243^ An example for achieving high loading efficiencies of proteins into polymersomes is to design specific interactions between the cargo and the polymersome membrane. Mertz and Castiglione genetically fused hydrophobic membrane anchoring peptides to the protein of interest and achieved more than 25% encapsulation efficiency during self-assembly.^244^ However, the proteins resided on the inside and the outside of the polymersome membrane, which required an post assembly processing step to enzymatically digest the protein on the outside surface of the vesicle.

Thus, encapsulation efficiency has to be thoroughly investigated for any new combination of cargo, block copolymer and polymersome preparation method. It cannot be assumed *a priory* that self-assembly of block copolymers in the presence of a cargo will yield loaded polymersomes, especially for the encapsulation of large molecules such as proteins and enzymes that will not diffuse through the polymersome membrane. Moreover, it should be checked if protein present in a polymersome preparation is actually inside of the polymersomes, or merely adsorbed to the surface of the polymersomes, for example by digesting surface-exposed proteins with proteolytic enzymes such as pronase or proteinase.^239^

The heterogeneous self-assembled structures produced by solvent exchange or film rehydration need homogenisation and purification steps in order to create polymersomes with a well-defined size. Size homogenisation is typically performed *via* extrusion through a track-edged membrane with defined pores through which the assembled structures are pressed. Typically, the pores of the membrane have dimensions of the order of nanometres to micrometres and function by reducing the dimensions of self-assembled objects that initially have a size that is larger than the pores. After numerous passages through the membrane, the structures typically have a more homogeneous size distribution and also have a smaller diameter, which is governed by the pore size. Attention must be paid to possible interactions of the polymersome with the membrane used, as possible absorption of the polymer can lead to a loss of polymer and lower the yield of polymersomes.

Once the polymersomes have the required size, impurities, non-encapsulated cargo, and agglomerates or other self-assembled structures need to be removed. This is typically done *via* dialysis, tangential flow filtration, size exclusion chromatography, or other types of chromatography. The basic principle underlying these methods is separation by size: polymersomes are much larger than small molecules or the polymers themselves, while micelles are typically also much smaller than polymersomes. A detailed discussion of these and other purification techniques can be found in previous articles.^240,245–247^

The successful homogenisation and purification ultimately need to be confirmed by detailed characterisation of the obtained polymersome solutions. Typically, a variety of techniques, including static and dynamic light scattering (SLS, DLS), TEM, and cryogenic transmission electron microscopy (cryoTEM) are used to characterize the structures. A detailed discussion of these different techniques is outside of the scope of this review and the different types of information gained from each technique are summarised in a previous review by Helix-Nielsen and coworkers.^248^

Even though solvent exchange and film rehydration, followed by extrusion, purification and characterisation are the most popular methods to prepare polymersomes, they suffer from several drawbacks such as their batch-to-batch variability, the broad size distributions of the resulting polymersomes, non-scalability, rather low encapsulation efficiencies, and lack of automation. As a result, these self-assembly methods represent hurdles to the clinical translation of polymersomes. The absence of an FDA- or EMA-approved polymersome-based therapy stresses the need for scalable and reproducible polymersome preparation methods in order to execute clinical trials and obtain reliable results.^249^ Moreover, reaching high encapsulation efficiencies or recovery of unencapsulated cargo is desirable from an economical point of view.^249^ Thus, novel techniques suitable for the self-assembly of polymersomes will have to be explored and implemented.

In the next section, we go beyond previous reviews on polymersome assembly techniques and highlight some newer vesicle formation techniques that enable a reproducible, fast, and large-scale production of polymersomes. Moreover, significant advances in the development of new liposome self-assembly technologies allow to circumvent the drawbacks of solvent exchange and film rehydration. Thus, novel trends from this field are outlined and their potential use for the formation of polymersomes is discussed.

### 5.2. Flash nanoprecipitation as polymersome self-assembly method

**Fig. 5:**
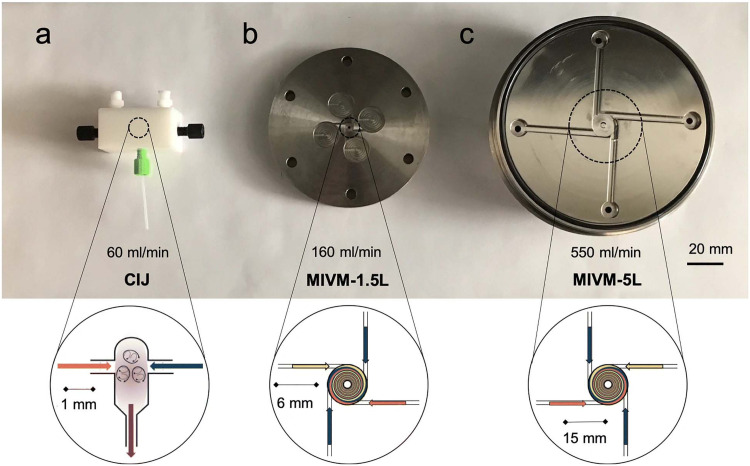
Photos and schematic drawings of (a) confined impingement jets (CIJ) mixer, and (b and c) multi-inlet vortex mixer (MIVM) for large scale production of polymersomes by flash nanoprecipitation. An aqueous phase (blue arrows) and an organic solvent phase containing polymers (orange and yellow arrows) are blended together to form polymeric vesicles. Reproduced with permission from J. Feng, *et al.*, *J. Transl. Med.*, 2019, **17**, 200 under Creative Commons CC BY license.^210^

Flash nanoprecipitation is a self-assembly method that was developed to gain more control over the nanoprecipitation process^250^ and which is widely used on a very large scale for the production of lipid nanoparticles for mRNA vaccines.^1^ For polymersome formation, an aqueous phase and an organic phase containing block copolymers are mixed by two confined impingement jets (CIJ mixer; Fig. 5), typically in a 1 : 1 ratio (Table 1).^209,210^ Subsequently, the mixture containing polymersomes is collected and passed through the CIJ mixer again and again, until the polymersomes are unilamellar and the size distribution is narrow.

Under laboratory conditions, the process is usually carried out manually with two syringes.^209^ Under typical conditions, the diameter of the resulting polymersomes varies between 65–300 nm, depending on the formulation,^211^ and their polydispersity index (PDI) is usually between 0.18–0.30, although values up to 0.63 have also been reported.^209^ Note, unlike the dispersity of polymers, which is always larger than 1, the PDI of particles is defined as the square of the standard deviation of the particle diameter distribution divided by the mean particle diameter and ranges from 0 to 1.^251^ Proteins can be encapsulated by adding them to the aqueous phase and have been shown to remain biologically active during the process.^209^ Typical encapsulation efficiencies of around 20% were reported for different hydrophilic compounds and upscaling is possible by using different mixers such as a multi-inlet vortex mixer (MIVM; Fig. 5),^210,211^ which enables mixing of larger volumes per batch. The MIVM mixer contains four inlets that are arranged perpendicular to each other and effuse into a central cavity where the different phases form a vortex in which they are mixed.

Flash nanoprecipitation has been used to prepare polymersomes for *in vivo* studies in mice and non-human primates.^212^ The main advantage of this method is the amenability towards upscaling, as it is expected that multiple kilograms a day can be produced by flash nanoprecipitation.^210^ However, the major drawbacks of this method are the relatively low encapsulation efficiency, the high fraction of organic solvent that is required, the high polydispersity of the samples under certain settings, and the requirement for multiple passages through the CIJ mixer to achieve uniformity.

Another method to improve control over the nanoprecipitation process involves the use of a stirred tank reactor (Table 1).^213^ In this process, an aqueous phase is stirred by a propeller, while a polymer solution in a water-miscible organic solvent is added by an external pump. The mixtures is continuously stirred beyond the point when the addition is complete. This results in an increase of the polymersome size during the first 25 minutes (from 30 to 180 nm) and a decrease in the PDI during the first 30 minutes (from >0.60 to 0.17), after which the size and PDI remain constant.^213^ Batches of up to 1.5 litres of uniform polymersomes were obtained with such stirred tank reactors at a final concentration of 10 mg mL^−1^ of polymer. Stirred tank reactors offer an improved reproducibility compared to manual nanoprecipitation processes, because of the semi-automated process, and the need for homogenisation by membrane extrusion is reduced. Size exclusion chromatography, dialysis or the like, are, however, still needed to remove the non-encapsulated cargo. A major drawback of this process is that it requires a large volume of an organic solvent, which is undesirable as it may denature therapeutic proteins and also poses safety and environmental concerns. Nonetheless, such reactors still reduce the volume of organic solvents to 5% of the total mixing volume, compared to 50% in the case of flash nanoprecipitation. On the downside, a stirred tank reactor mixes the aqueous non-solvent and organic solvent with a turbulent flow, *i.e.* at high Reynolds number (Re), which lowers the level of control that can be exerted over the mixing process. Also, particle agglomeration may take place during the procedure. As a consequence, polymersomes with a very narrow size distribution (PDI < 0.10) cannot be obtained without additional homogenisation, purification and, as a result, significant loss of polymersomes.

### 5.3. Microfluidics as polymersome self-assembly method

**Fig. 6:**
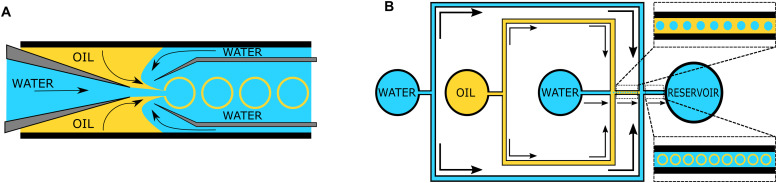
Schematic depiction of microfluidic water-in-oil-in water double emulsion techniques to form giant polymersomes (and other microcapsules). (A) One-step microcapillary method. The central water phase flows through a capillary into the oil phase, that then flows into the outer oil phase and a collection capillary. To form giant polymersomes, the oil phase would contain amphiphilic block copolymers, that then self-assemble into vesicle membranes. (B) Two-step chip-based method. In the first step, a single emulsion is created in which droplets of the inner water phase are suspended in the oil phase. Then, this emulsion flows into the second aqueous phase, forming double emulsion droplets. If the oil phase contains suitable block copolymers, giant vesicles can be obtained.

The disadvantages of mixing at high Re number, *i.e.* particle agglomeration, can be circumvented by using microfluidic devices with tubing of small cross-sectional areas in which viscous forces dominate and flow is laminar, *i.e.*, under conditions at which the Re is low.^252^ A laminar flow prevents self-assembled nanostructures from interacting or coalescing with each other, thereby preventing additional particle processing during or after the self-assembly step.^253^ Double emulsions, namely water-in-oil-in-water (w/o/w) double emulsions, are one of the most widely used microfluidic approaches to produce polymersomes.^218^ Such emulsions are most commonly formed in capillary microfluidics (Fig. 6A) or in microfluidic chips (Fig. 6B) by flowing a droplet of solvent through a succession of different, immiscible solvents. The final w/o/w double emulsions feature core-shell structures with a hydrophilic core and a hydrophobic polymeric membrane inside a hydrophilic solvent. Capillary and chip-based methods are based on the same principles. However, the set-up differs slightly between the two methods. More in-depth analyses on microfluidic methods are available in the literature.^218,254,255^ In general, the control and reproducibility is better with capillary methods than microfluidic chips.^218^ The main advantages of double emulsions are that they afford highly uniform polymersomes (PDI < 0.10) and that a negligible quantity of other nanostructures (*e.g*. polymeric micelles) is obtained. Although the size can be controlled to some degree, double emulsions are mainly interesting for the self-assembly of polymeric vesicles on the micrometre-scale (*i.e*. giant polymersomes). This technique is already being used to assemble polymersomes and liposomes. A recent study in which hybrid lipid-polymer vesicles were prepared by a w/o/w emulsion and which were used to encapsulate gold nanorods, composite nanoparticles, DNA, antibodies, and hydrophobic drugs highlights the versatility of the double emulsion process.^256^ Furthermore, high protein encapsulation efficiencies (up to 100%) can be reached with w/o/w double emulsions by simply dissolving the protein in the inner phase prior to the self-assembly process.^216,257^ However, double emulsion microfluidics are mainly used by a select number of groups because the process requires highly specialised equipment which, although commercially available,^258^ still needs to be handled by an experienced user.^218,254,255^ Most microfluidic double emulsion methods result in micrometre-sized vesicles, whose dimensions limit their suitability for nanomedicine applications. Thus, these methods are mostly interesting for researchers who anticipate to be focusing on giant polymersomes or other microspheres on the long-term, as only then it might be worth to invest the time and resources to acquire and master such a self-assembly set-up.

**Fig. 7:**
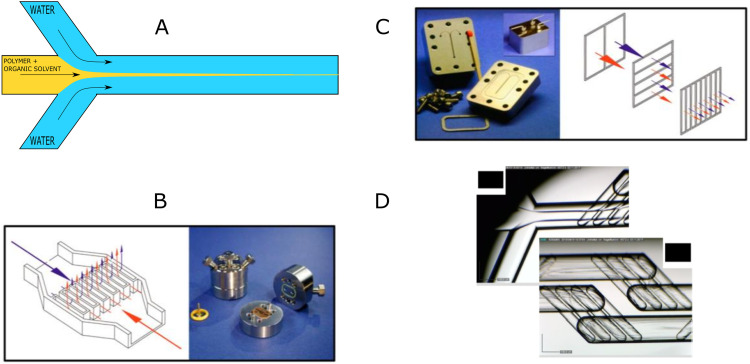
Hydrodynamic flow focusing and microfluidic micromixers for the preparation of polymersomes and other self-assembled structures. (A) Schematic illustration of hydrodynamic flow focusing under a 45° angle. (B) Interdigital micromixer pattern and corresponding micromixer device. (C) Caterpillar micromixer pattern and corresponding micromixer device. (D) Commercially available Dolomite microfluidic device. Images B and C adapted with permission from R. Bleul *et al.*, *Macromolecules*, 2015, **48**(20), 7396–7409.^259^ Copyright 2015 American Chemical Society. Image D adapted with permission from L. Albuquerque, *et al.*, *Langmuir*, 2019, **35**(25), 8363–8372.^260^ Copyright 2019 American Chemical Society.

Another type of microfluidic technique that has been used for polymersome assembly, and which is already established for liposome assembly, is hydrodynamic flow focusing. In this process, a central stream of an organic solvent containing the amphiphilic molecules, such as block copolymers or lipids, is accompanied on both sides by an aqueous flow (Fig. 7A).^215,255^ Control can be exerted by changing the fractional flow rate (FFR), the total flow rate (TFR), and the copolymer concentration.^259^ The FFR is the ratio of aqueous to organic phase administered into the system, and can be used to control the size of the particles.^215,255^ Higher FFR's, *i.e.* more diluted flows, result in smaller polymersomes, whereas lower FFR's, *i.e.* more concentrated flows, yield larger particles. The aqueous phase is usually added to the organic phase at a 90° or 45° angle. The input parameters, such as the TFR and FFR, are easy to control *via* an electronic interface and appropriate pumps, which reduce the error margin of this approach. The precisely controlled flow creates an interface between both phases at which the polymersomes are predominantly formed. Polymersomes will also form in the central stream further down the tubing once the organic phase is sufficiently diluted. Unfortunately, encapsulation efficiencies are low, since only a minor fraction of both solvents is directly involved in the polymersome self-assembly at the interface. For example, bovine serum albumin protein was encapsulated at an efficiency of 29%.^217^ Nonetheless, the resulting polymersomes were highly uniform (PDI: 0.05–0.20) and had a controllable diameter between 40 nm to 2 μm.

Micromixers form the basis of another microfluidic technique, in which a central element with a particular mixing pattern mixes the aqueous and organic phase. Micromixers provide excellent control over the size of the polymer vesicles, which can be tuned by the FFR, TFR, copolymer concentration, and the type of mixing element. An organic polymer solution and an aqueous non-solvent are guided through microfluidic channels into a central element that mixes both phases. This leads to a drastic increase of the interfacial surface, which, in turn, leads to an increase in diffusion and precipitation of individual polymers chains into nanostructures. The key aspect of this technique is the mixing pattern, with well-known ones including the interdigital pattern (Fig. 7B) and caterpillar pattern (Fig. 7C).^259,261^ Just as other micromixers, these two micromixers improve the polymersome formation efficiency by increasing the rate of diffusion of polymers and solvents, which in turn is enhanced by the drastic increase in surface area between both phases. Consequently, the nanoparticle formation at the interface is significantly enhanced.

A commercial micromixer method was recently investigated for its capability to enable polymersome assembly. The system consists of a microfluidic cartridge (Fig. 7D).^260^ Similar to other micromixers, it facilitates precipitation of block copolymers into nanoparticles by highly efficient mixing of the organic and aqueous phase by creating lamination of the flow streams.^262^ In one study, the micromixer yielded polymeric nanostructures with a hydrodynamic radius of between 22 nm and 85 nm.^214^ The authors argued that nanostructures with a radius ≥50 nm corresponded to polymersomes, while smaller objects most likely were micelles. PDI values varied between 0.04–0.10, which indicated highly uniform polymersomes. In another study, polymersomes with diameter between 134 and 180 nm were obtained with a very low PDI of 0.05 to 0.06.^260^ A major advantage of these commercial micromixers is that the flow channels are made of glass, which ensures compatibility with a wide range of organic solvents.^260^ Furthermore, it is possible to choose between a hydrophilic or hydrophobic coating, and up to three different fluid streams can be mixed. A drawback of these micromixers is the production rate of self-assembled products, which is only in the order of a few μL min^−1^.

In general, micromixer-assisted nanoprecipitation processes offer two main advantages over manual nanoprecipitation: automation and reproducibility. The process is not user-dependent, and is repeatedly executed under the same controlled conditions. Moreover, microfluidic-based nanoprecipitation occurs in a laminar flow, which strongly reduces the interactions between formed nanostructures. Therefore, the polymersomes are more reproducible and highly uniform in size (PDI 0.05–0.20; Table 1). Interestingly, a non-microfluidic mixing set up was also able to provide a laminar flow and self-assembled quantities of more than 3 kg day^−1^.^219^

In summary, microfluidic approaches for polymersome self-assembly have not been widely adopted within the community. Polymersome self-assembly by hydrodynamic flow focusing has been described a decade ago,^215^ and despite its simple and inexpensive set-up it has been rarely used since its first description. Micromixer-based self-assembly has also not been routinely used for polymersome preparation, despite the proven ability to prepare highly defined nanostructures. Several reasons may be at the root of this, with the most prominent being an unawareness of these techniques within the polymersome community, which we aim to remediate with this review. Further, the misapprehension that the fabrication of such devices requires expertise in specific manufacturing methods, such as (soft)lithography,^215^ may also be a deterrent. While this is somewhat true for double emulsion microfluidic approaches, 3D-printing and downloadable designs for microfluidic devices have facilitated their use. Furthermore, the number of materials suitable for microfluidic chips has greatly expanded from the initial use of PDMS, which is not compatible with commonly used solvents such as tetrahydrofuran.^218^ Microfluidic micromixer devices made of glass can now be found commercially.^214,258,260^ In the end, the most likely explanation is that scientists tend to focus on the design of new polymersomes rather than taking upscaling amenability into account. Yet, we are convinced that such continuous manufacturing methods offer a valuable advantage to research groups, as it facilitates polymersome assembly while increasing the output, speed, reproducibility, and control over the self-assemblies. This saves valuable time by avoiding additional membrane extrusion steps, or repetition of experiments due to batch-to-batch variations. Furthermore, it is easy to screen for the optimal conditions to self-assemble polymersomes with microfluidics since the parameters are highly controlled, can easily be altered, and the production of polymersomes is fast. The protein encapsulation efficiency depends significantly on the used microfluidic technique with high encapsulation efficiencies for double emulsions (>84%) and lower efficiencies for the hydrodynamic flow focusing method (29%).^216,217^ Commercial systems are available, and set-ups can be easily self-fabricated using inexpensive materials.^214,219^ Nonetheless, the relative large fraction of organic solvent (5–65% vol%),^215^ which has a strong effect on the polymersomes’ size and PDI, may be disadvantageous in industrial upscaling.^216,217^ One could argue that a fraction of organic solvent can be recovered post-assembly and re-used to assemble another batch of polymersomes, at the expense of set-up complexity. It is also unclear how solvent recycling affects the self-assembly process.

### 5.4. Polymerisation-induced self-assembly (PISA)

**Fig. 8:**
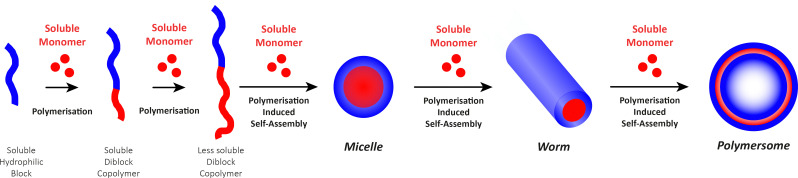
Schematic representation of the polymerisation-induced self-assembly (PISA) process.

While all of the above methods have been successfully transferred from the liposome field to the polymersome field, polymerisation-induced self-assembly (PISA) is a recently developed technique that is unique to polymer self-assembly.^220,263^ The principle of the process is the use of a water-soluble macro chain-transfer agent (*e.g.* a poly(ethylene glycol)-based reversible addition-fragmentation chain-transfer (RAFT) agent) or a hydrophilic macromolecular initiator to polymerise a water-soluble monomer into a polymer that becomes water-insoluble as the degree of polymerisation increases (Fig. 8).^220,264^ The resulting amphiphilic block copolymers thus precipitate during the polymerisation process into nanostructures.^220,264^ By fine-tuning the degree of polymerisation of the hydrophobic block and, therefore, the hydrophilic-to-hydrophobic block ratio, assemblies ranging from micelles over worms to polymersomes can be produced.^264,265^ One of the features that makes PISA unique is the ability to perform two steps at once: the polymer synthesis and the assembly of the polymersomes. Moreover, the assembly step can take place at high polymer concentrations of up to 50 wt%.^220^ Protein encapsulation efficiencies of as high as 79% have been reported,^222^ although typical encapsulation efficiencies remain in the range of 9–27%.^220,264^ Typical polymersomes prepared by PISA are between 230–760 nm in diameter, and display a narrow size distribution (PDI 0.04–0.20, Table 1). RAFT polymerisation is by far the most widely used polymerisation technique for PISA, but other methods such as atom-transfer radical polymerisation (ATRP), and nitroxide-mediated radical polymerisation (NMP) have also been explored.^220,263^ Several in-depth reviews are available that detail these processes and their mechanistic details.^220,264^ The polymerisations are rather fast and can be performed in aqueous dispersions under mild conditions, which are generally suitable for protein encapsulation. This is a unique feature of PISA, since other methods solely reduce the amount of organic solvent, but do not avoid it. Nevertheless, there are several factors that limit the applicability of PISA-based polymersomes for clinical applications. The most important problem is the difficulty to remove unreacted monomers from the assembled structures. One could argue that the concentrations of unreacted monomer are, in general, fairly low, since the monomer consumption is close to full conversion, yet this remains only an assumption.^264^ Even though non-encapsulated monomer can easily be removed post-assembly by either dialysis or centrifugation, there is no general protocol to remove unreacted monomers that are encapsulated within the nanostructures,^224,266^ and little is known about their effect on cells.^264,266–268^ This poses great challenges in clinical validations, as the exact amount of residual monomer is not known, especially in light of the toxicity of many frequently employed monomers.^268^ Another important drawback of PISA is that the encapsulation efficiency is, in most cases, lower than that of some of the highly efficient microfluidics-based techniques.^268^ Moreover, PISA is typically performed on a small scale (batches <20 mL).

### 5.5. Conclusion: existing polymersome preparation methods

To conclude, self-assembly methods such as solvent exchange (*i.e.* manual nanoprecipitation) and thin film rehydration are simple to use but somewhat outdated. Not only do these methods offer low control over the size of the vesicles, but the polymersomes produced suffer from a broad size distribution (PDI > 0.2) and the encapsulation efficiencies are low.^208^ Even though these techniques can be considered convenient because of the minimal equipment requirements, many alternatives are commercially available, relatively cheap,^214,258^ and lead to much more homogenous structures and, oftentimes, higher encapsulation efficiencies. Flash nanoprecipitation and stirred tank reactors are interesting as they are able to produce large batches of polymersomes with a narrow size distribution and reasonable encapsulation efficiencies (up to 43%).^209,210,213^ Microfluidic methods, *e.g.* double emulsions and hydrodynamic flow focusing, yield highly defined vesicles (PDI < 0.10), and can reach impressively high encapsulation efficiencies. It is important to consider which microfluidic method is the most suitable for a specific application. For instance, capillary double emulsions are promising if a very high encapsulation efficiency is wanted, yet these droplet-based approaches would not be the first choice if it is crucial that the vesicles are <100 nm in size. Another major drawback is that the output of microfluidic devices is usually in the order of μL min^−1^. PISA is a relatively new assembly method that facilitates simultaneous polymer synthesis and assembly into nanostructures.^222^ The main disadvantages of PISA are possible toxic effects of unreacted monomer that have not been thoroughly examined *in vivo*.^266–268^ Another possibility that was not discussed in-depth is the possibility to combine various techniques. A powerful example of this is the combination of a continuous flow reactor set-up for PISA,^221^ allowing facile upscaling to 60 grams of nanostructures per day, while forming highly uniform and reproducible samples.

### 5.6. Future polymersome preparation methods: Inspiration from liposome preparation

A plethora of preparation methods for liposomes exist and multiple previous reviews discuss these.^255,269–271^ Liposomal self-assembly methods outnumber the polymersome self-assembly methods, and continuous manufacturing methods are more frequently employed for liposomes, resulting in more reproducible results and high encapsulation efficiencies. Furthermore, the assembly methods are more advanced in terms of scalability, time-efficiency, overall complexity, and sometimes even avoid the use of organic solvents. In the previous section, we discussed some of the most advanced methods for homogeneous and reliable preparation of polymersomes. Most of these techniques have been adopted from the lipid field, and it appears that state-of-the-art methods employed for liposome, niosome and exosome assembly may also be adaptable for the production of polymersomes. Herein we focus on liposomal self-assembly methods which are single-step, scalable, fast, and ideally avoid the use of organic solvents.

**Fig. 9:**
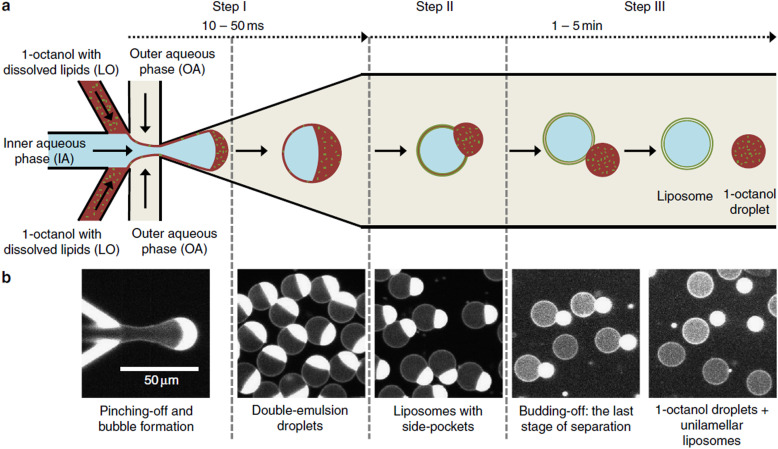
Octanol based liposome self-assembly. (A) Schematic overview of the microfluidic chip-based double emulsion liposome assembly process. (B) Fluorescence microscopy images showing the structure of the droplets and liposomes at each step. Reproduced with permission from S. Deshpande *et al.*, *Nat. Commun.*, 2016, **7**(1), 10447 under Creative Commons CC-BY licence.^229^

Compared to the above-described double emulsions technique, a more advanced octanol-based assembly method on a chip has been reported in the context of liposome assembly (Fig. 9).^229^ As in standard double emulsion techniques, the lipids are dissolved in an oil phase, 1-octanol, which is then passed through a microfluidic chip with the inner and outer phase being aqueous mixtures (Fig. 9). The vast majority of lipids dissolve easily in 1-octanol. The lipids form a membrane around the central aqueous phase while the octanol phase distributes itself asymmetrically around the droplet, eventually leading to the formation of an octanol pocket. Thus, excess lipids and the octanol phase are removed concomitantly from the self-assembled lipid bilayer. Finally, the octanol droplet that contains excess lipid pinches off, thereby leaving a unilamellar liposome behind. This method can be used to self-assemble micrometer-sized liposomes (5–20 μm) with a very narrow size distribution (coefficient of variation of 4–11%). The coefficient of variation (or standard deviation) is a way to describe the size distribution of nanoparticles and corresponds to a PDI 0.05. This technique yields solvent-free liposomes, and octanol droplets that can be separated in-line.^230^ This paves the way for solvent-free liposome formulations, while using the benefits of solvent-assisted assembly.^230^ Though the total absence of octanol in the membrane of the liposomes cannot be guaranteed, any minor solvent traces are not expected to induce significant toxic effects due to the relative biocompatible nature of 1-octanol.

**Fig. 10:**
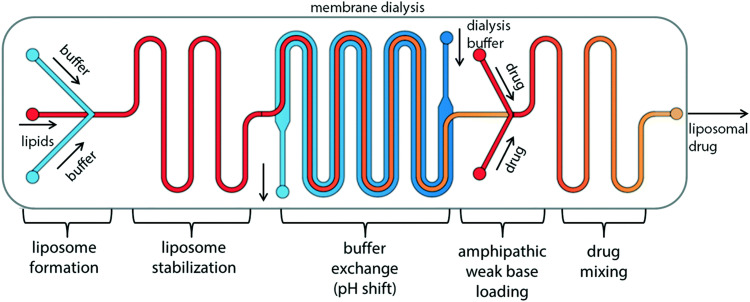
Schematic showing the integration of liposome self-assembly, purification, and post-assembly drug loading on a single microfluidic chip. Liposomes are formed by hydrodynamic flow focusing and the buffer is exchanged by counterflow membrane dialysis to create a transmembrane ion and pH gradient. Then, the liposomes are loaded with drugs by remote loading. To this end, an amphiphilic drug diffuses from the outside through the vesicle membrane into the vesicles where it encounters high salt concentrations and/or acidic pH, which causes the payload to be trapped inside of the vesicles. Reproduced from R. R. Hood, *et al.*, *Lab Chip*, 2014, **14**(17), 3359–3367 with permission from The Royal Society of Chemistry.^227^

Microfluidic self-assembly of liposomes can also be integrated together with post-assembly purification and drug loading on a single microfluidic chip, as demonstrated by Hood *et al.* (Fig. 10). The authors assembled, purified and loaded polymersomes within a three minute time frame through hydrodynamic flow focusing.^227,272^ The assembled liposomes displayed very narrow size distributions (PDI: 0.05), and had a controllable size between 190 and 225 nm.^227^ Purification was accelerated by counterflow microdialysis, a process in which buffer flows alongside the liposome channel in the opposite direction.^227^ The channels were separated by a porous membrane that enabled ion exchange and prepared the vesicles for the next step, the encapsulation of drugs by remote loading. The latter is a technique in which amphiphilic drugs such as DOX are loaded into assembled liposomes by using a pH or ionic gradient across the vesicle membrane.^273^ The uncharged payload diffuses through the membrane into the vesicles, where an appropriate low pH leads to the protonation of the compound, so that the positively charged drug cannot diffuse back through the membrane. Alternatively, high salt concentration in the vesicles can lead to precipitation of the drug in the vesicles. Both processes accumulate the payload in the vesicles. Encapsulation efficiencies were around 70%.^227^ Recently a similar, easily scalable microfluidic system was described, which afforded drug-loaded liposomes in under two minutes, showing that even shorter timeframes for self-assembly are achievable.^228^ The liposome's size could be tuned between 60 and 800 nm.

To conclude, liposomes can be self-assembled, purified, and loaded post-assembly on a single microfluidic chip in a reproducible manner. Integration of these processes on a single device offers a swift and reliable self-assembly process, which is of interest since the most frequently employed polymersome self-assembly techniques often take hours to days to self-assemble, purify, and load polymersomes with biological agents. Remote loading is most likely not applicable to proteins since their stability and biological function changes with changes in pH and ion concentration. Nonetheless, protein encapsulation could still be possible but at a low encapsulation efficiency.^217^

With the goal of upscaling liposome production, the Perrie group recently investigated microfluidic mixing devices with a toroidal mixer design that is able to self-assemble up to 20 L of unilamellar liposomes per hour.^225^ Liposome self-assembly is facilitated by toroidal mixing, which enables self-assembly by vortices and centrifugal forces, inducing chaotic advection while preserving laminar flow. The size of the liposomes could be tuned between 50–175 nm by altering the FFR, lipid type, and lipid concentration. Furthermore, the assembled vesicles had narrow size distributions (PDI 0.07–0.19). Typical values of protein encapsulation efficiencies were usually between 20–35%, although values as low as 14% were also reported by the same group.^225^ On a side note, encapsulation efficiencies of an RNA surrogate reached values between 95–100% when employing cationic lipids.^225^ Interestingly, the device also allows small batch production of liposomes (1 mL) by simply changing the TFR, without requiring any additional changes of the process parameters. This easy translation of small-scales to large batches enables a smooth transition from bench to production-scale.

Swift assembly and scale-up was also achieved in a device that assembles, homogenises, purifies, and sterilises liposomes in one continuous flow.^274^ A phospholipid organic solvent mixture was heated to 80 °C and passed through a thermal mixing device. The mixture flowed through an aseptic filter in the mixing device which sterilised the solution. Subsequently, the mixture was cooled to 20 °C and liposomes start to assemble. The cooled mixture was then purified by counterflow dialysis to remove any residual organic solvent. No endotoxins, trace metals, or bacteria were detected after the entire process. The assembled liposomes varied between 70–140 nm in size and were monomodal with a PDI between 0.06–0.13, although a PDI of 0.21 was observed in one scenario. Upscaling up to a volume of 4 L was described, and the manufacturer claims that an encapsulation efficiency of hydrophilic compounds between 40–60% can be achieved.^275^ The question for protein nanocarriers remains whether the passage through the heating element would cause protein denaturation or whether the device allows introducing the protein solution during the cooling stage.

**Fig. 11:**
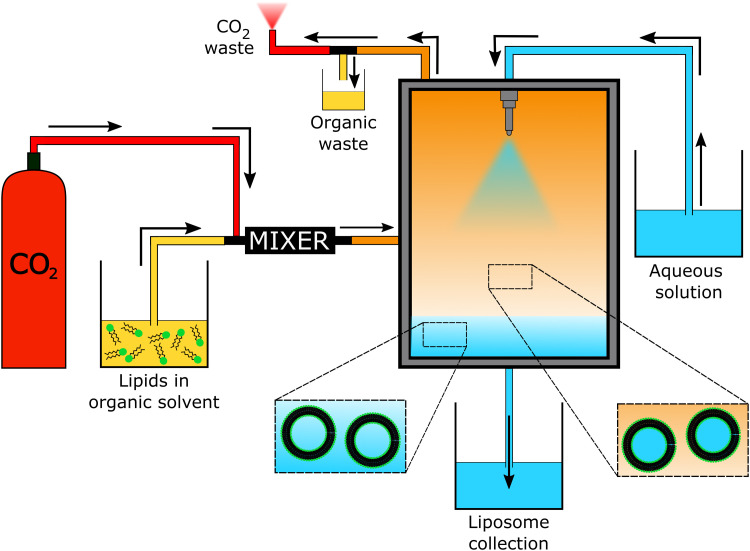
Schematic set-up of the Supercritical Assisted Liposome formation (SuperLip) method to self-assemble liposomes. A solution of lipids in organic solvents is mixed and expanded with supercritical CO_2_ in a pressure chamber. Water droplets, containing *e.g.* proteins, are formed by nebulisation in that chamber, so that the liposomes form around them. The aqueous vesicle suspension accumulates at the bottom of the chamber and can then be retrieved.

Reports on organic solvent-free preparation methods are limited but they are increasingly receiving attention. There are some existing, cargo-specific approaches that avoid the use of organic solvent by *e.g.* pH-driven loading of agents into liposomes.^276^ Even though this method can be easily scaled up, it is not widely applicable to proteins and other macromolecules. Supercritical fluids (SCF), on the other hand, have the potential to reach high encapsulation efficiencies while avoiding the use of organic solvent, thereby offering a greener alternative to encapsulate proteins and other sensitive compounds.^277^ Supercritical CO_2_ is the most commonly used SCF for liposomal self-assembly (Table 1)^278–280^ and can be used as a solvent, co-solvent, anti-solvent, or dispersing agent.^280^ One method that uses SCF as a co-solvent, the SuperLip process (supercritical assisted liposome formation), has received a lot of interest since extraordinary high encapsulation efficiencies can be reached (92–98%).^231,280–282^ In this process, the lipids are first dissolved in an organic solvent, which is subsequently mixed with a supercritical phase to create an expanded solution inside a high-pressure vessel. An aqueous solution, *e.g.* containing proteins, is then nebulised into the vessel (Fig. 11). Almost instantly, the lipids in the SCF phase form a bilayer around the aqueous droplets, and the resulting liposomes accumulate in water at the bottom of the high-pressure vessel. Finally, the liposome solution is collected in a vial, the organic solvent is collected in a waste bottle, and the supercritical CO_2_ is vented out. Unfortunately, a considerable fraction of the total solvent volume (SCF + organic solvent) still consists of organic solvent (29 wt%) for this SuperLip method.^231^ Other SCF-based assembly methods require a lower fraction of organic solvent, *e.g.* 7 wt%, at the cost of a lower encapsulation efficiency (down to 73%).^283^ In this case, self-assembly was facilitated by simply dripping the aqueous phase into the SCF phase, while stirring. Liposomes prepared by this method were more stable than ones assembled by the thin film rehydration method, which was assessed by comparing the retention of BSA during storage. Self-assembly by SCF-based methods is not only faster and more scalable than the thin film rehydration method, but it also yields more uniform products (PDI: 0.17–0.20) between 166–305 nm in diameter. Large batch production is achievable by commercially available systems but notable drawbacks of this technique are the level of expertise and specialised equipment needed, as well as the dangers that are associated with supercritical fluids. Related risks include high pressure, dry ice formation, explosions, and asphyxia in case of leakage.

The heretofore mentioned examples are just a few of the many interesting self-assembly methods that have recently been reported to produce liposomes. It is clear that continuous manufacturing has taken up a fortified position because of its amenability to upscaling, the time-efficiency, and the production of highly uniform self-assemblies with a tuneable size. In contrast, microfluidic self-assembly methods are seldomly employed in the polymersome field, especially when double emulsions are not taken into account.^214,256,284^ Furthermore, most of the previously mentioned techniques can easily be performed on minimal equipment, and more complex systems can be self-made with commercially available, inexpensive products. Admittedly, some methods (*i.e.* supercritical fluids) require specific expertise and equipment, but collaborations or a cooperation with an industrial partner may allow to overcome this hurdle. Of course, translating each individual technique towards polymersome self-assembly will bring along its own challenges. Especially the choice of solvent can be quite challenging since many commonly used polymer solvents (*e.g.* tetrahydrofuran, dichloromethane) may not be compatible with the tubing or the commercial micromixer cartridges used for liposome assembly. This means that polymers may have to be dissolved in a solvent compatible with the device, or the assembly devices will have to be built with tubing, materials, and seals that are compatible with harsher organic solvents. Nevertheless, there are plenty of excellent assembly methods that have been developed for liposomes and wait to be exploited for the production of polymersomes.

## 6. Protein delivery with polymersomes: barriers for cellular uptake

Once formed, loaded with therapeutic cargo, and purified from non-encapsulated drugs or proteins, polymersomes must be administrated through a suitable route. Many administration routes are possible and depend on the final target of the therapy. Invasive, parenteral administrations, such as intravenous or intramuscular injections, are some of the most commonly used administration types for nanomedicines, as it allows the therapeutic drug to bypass physiological barriers, *e.g.* the digestive tract, that could otherwise compromise its bioavailability.^285,286^ Likewise, other post-administration biological barriers within the human body must be considered during the engineering of therapeutic nanocarriers, since they dictate the fate of the drug delivery.^285^ Imposed as natural checkpoints against potentially harmful agents, these biological barriers primarily include the rapid clearance by the reticuloendothelial system (RES), as well as endothelial and cellular barriers, among others.^287,288^ These biological barriers can severely limit the mode of action of a given therapeutic nanocarrier, potentially affecting its time in circulation, biodistribution, drug bioavailability at the site of action, cell uptake and drug release, as well as biodegradation, thus hampering the expected therapeutic efficacy. All of these variables need to be faced in order to allow for successful drug and, by extension, protein delivery.^289^ More importantly, the complexity and influence of each barrier will naturally depend on the specific setting in which they are encountered. Factors such as the type of nanocarrier, disease, or targeted tissue play a determinant role. The reader is directed elsewhere for comprehensive reviews on the topic.^287,288,290^ Conversely, to establish a frame of work, the focus of this chapter will be on the biological barriers addressed and tackled by relevant polymersome-based protein delivery strategies.

In the context of designing polymersomes for therapeutic protein delivery, the main distinguishing features of polymersomes are the different approaches to overcome one or a combination of several biological barriers, commonly being the rapid clearance by the reticuloendothelial system, also known as mononuclear phagocytic system (MPS), cellular internalisation, and intracellular protein release through endosomal escape. It is important to note that, depending on the therapeutic objective, reports of therapeutic polymersomes address these barriers differently, especially since the complex and dynamic environment found in the body renders cellular uptake a unique challenge to tackle, due to the wide range of concomitant and intricate factors ruling the process.^291^ Therefore, the examples addressed in the following sections are meant to highlight significant advances of polymersome-based protein delivery formulations in crossing such barriers. Comparisons and parallels with the liposome literature will be made in order to highlight challenges within the polymersome field which need to be addressed for successful translation of polymersome-based delivery systems into clinical trials.

### 6.1. Reticuloendothelial system (RES) clearance

Especially in the case of systemic circulation, phagocytic macrophage bodies from the spleen, liver and lymph nodes, constituting the RES, act towards the removal of blood-borne particulates. Once a given nanotherapeutic is introduced into the biological environment, it is approached by a complex cornucopia of biomacromolecules. A protein corona is formed on the surface of nanocarriers after administration, which leads to recognition and uptake of these nanoparticles by the phagocytes.^292^ The formation of this protein coating ultimately influences the nanocarrier's uptake path,^293,294^ which is known to be dependent on size, hydrophobicity, surface chemistry, and charge of the nanocarriers.^292,295–298^ Moreover, it is also strongly influenced by the specific environment in which it occurs, making it difficult to predict.^299^ Consequently, together with uptake and clearance by the RES, opsonisation substantially limits most active-targeting capabilities.^295,300,301^ The attachment of poly(ethylene glycol) (PEG) to the surface of polymersomes or the use of PEG-based amphiphilic block copolymers prevents this problem, as it prevents protein adsorption and thus “camouflages” the nanocarrier. The wide availability and biocompatibility of PEG has made it the gold standard for polymersome-based protein delivery systems. While this effectively minimizes their premature RES clearance from circulation, thereby increasing their circulation half-life,^302–304^ there is still a debate over the potential immunogenicity of PEG-based formulations that affect the very advantages associated with their use.^99–101^ For instance, studies have shown that PEG can potentially reduce interactions with the target cells,^305,306^ and also be prone to anti-PEG immune responses that may occur upon repeated administration, and elicit clearance from circulation.^305–307^ Therefore, other polymeric materials have been reported as PEG-alternatives that include polysaccharides,^308^ peptides,^309^ poly(2-oxazoline)s (POx),^310^ poly(phosphoesters) (PPEs),^311^ and poly(zwitterions).^312^

In addition to the use of PEG or its polymeric alternatives, other methods to avoid RES clearance have been reported for liposomes and other nanocarriers, but not for polymersome-based protein delivery. Such stealth alternatives include the surface functionalisation with “don’t-eat-me” peptides,^313–315^ which are markers that bypass phagocytic activity from the RES and have been shown to significantly reduce RES clearance. Similarly, the functionalisation with membrane extracts of red blood cells (erythrocytes), white blood cells (leukocytes),^316^ or platelets (thrombocytes),^317^ have also been attempted. With the same goal, nanocarriers have been decorated with natural proteins, such as albumin, alipoprotein, or dysopsonins.^309^ Akin to the use of natural stealth polymers, these PEG alternatives avoid the immunogenicity issues that are associated with PEG.^309^ However, as with any interplay between synthetic and biological components, biological sensitivity and function must be considered and a full understanding of the interplay between phagocytes and nanoparticles is needed. These strategies are, up to date, largely unexplored for polymersomes.

Insulin is a prime example of a therapeutic protein. Looking at insulin delivery more closely, current strategies to keep blood sugar levels constant through the prolonged presence of insulin in the blood stream rely on repeated subcutaneous injections of this protein. Encapsulation of insulin within a nanocarrier allows to substantially increase its circulation times,^318,319^ as the PEG corona suppresses the RES clearance. PEG-based polymersomes have been described to improve insulin delivery in the literature.^320–323^ Most reports show improved blood sugar levels in *in vivo* studies with a prolonged effect compared to injected bare insulin. In addition, the structural flexibility of polymers, the high structural stability of polymersomes,^4,324^ and the ability to create polymersomes that release insulin in response to stimuli,^325,326^ provide polymersomes with inherent advantages over liposomes. Nonetheless, liposomes are still more established with regards to insulin delivery strategies. Moreover, typical shortcomings of liposomes compared to polymersomes could potentially be reduced by improving liposome stability, functionalisation of the liposome surface with sugar recognising moieties, and with permeation-enhancing molecules to facilitate translocation from the gastrointestinal tract to the blood stream.^327–330^

In contrast to insulin delivery, many other forms of therapeutics rely on taking advantage of RES's uptake to trigger immunogenicity, the typical case being vaccination. In this regard, polymersomes provide suitable antigen delivery properties because of their pathogen-like structure (robust and high molecular weight units), and because they are able to carry bioactive protein antigens and other small molecules necessary for generating an immune reponse.^331^ These adjuvants are key in attaining immunogenicity as they help the amplification of immune responses, form antigen depots at the injection site, protect antigens against enzymatic degradation, or activate and deliver antigens to antigen presenting cells (APCs), such as dendritic cells (DCs) or macrophages.^332–334^ Classical vaccine adjuvants like aluminum salts or Freund's adjuvants (bacterial antigen emulsion) are commonly used,^334,335^ but these have come under scrutiny for their local and systemic toxicity. Polymersomes could provide a suitable alternative to classical adjuvants. Early work involving the capability of polymersomes to enhance immunogenicity provided evidence of the polymersomes’ ability to enhance the immunogenicity of an influenza subunit vaccine.^336^ The study employed a hydrophobic poly(γ-benzyl-l-glutamate) block and a short peptide as the hydrophilic block^337^ which complexed with the hemagglutinin antigen (HA) through hydrophobic and electrostatic interactions, respectively.^336^*In vivo* tests showed that HA/polymersome complexes enabled up to a twenty-fold increase in the hemagglutinination inhibition, *i.e.*, immune response, compared to the non-adjuvanted/free form of the hemagglutin antigen. The adjuvant-like feature of polymersomes was theorised to be related to their ability to provide an antigen depot, thereby increasing antigen uptake by the APCs which, akin to classical adjuvants, triggers a more pronounced immune response.^338^

**Fig. 12:**
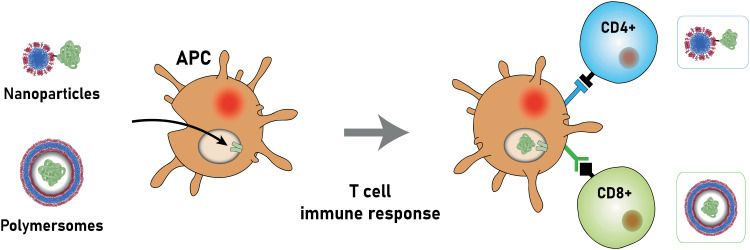
Immunisation with polymer nanocarriers *via* endosomal antigen receptors of activated antigen presenting cells (APCs) (Hubbell and coworkers^340–343^). Depending on nanocarrier morphology, as well as on the transportation mode of the antigen (encapsulation *vs.* surface functionalisation), different types of T cell immune response may be elicited: once captured, the nanocarrier releases its antigen (cargo) into antigen-specific receptors, located within the activated endosomal compartments of the APCs. The appropriate immunisation pathway is then triggered, *e.g.* predominant recruitment of either CD4+ or CD8+ T cells, depending on the nanocarrier type.

Work by Hubbell and colleagues on neonatal tuberculosis vaccination aimed at using DCs, a subset of APCs crucial in developing immunogenicity, as a means to enhance innate as well as adaptive early-life immune responses in mice.^339^ To emulate the nanostructural diversity of viruses, the authors firstly compared micelles, filomicelles, and polymersomes made of an oxidation-responsive PEG-*b*-PPS block copolymer. *In vivo* biodistribution assays indicate that the selective targeting and uptake of different types of DCs was highly dependent on the morphology of the nanocarriers, with polymersomes being able to target more subgroups of DCs in comparison to micelles and filomicelles. Conversely, polymersomes co-encapsulating a *M. tuberculosis* antigen, and a poorly soluble and systemically toxic dendritic cell agonist (endosomal TLR8/7) triggered adult-like antigen-specific T cells immune responses in neonatal humanized mice. The immune response was comparable to the already administered tuberculosis Bacillus Calmette-Guérin (BCG) vaccine. Hubbell and colleagues also described the polymersome-based delivery of ovalbumin (OVA) antigen aimed at endosomal cross-presentation receptors of DCs^340–343^ that, once activated, initiate a series of events required for the priming of T cell antigen-specific immune responses (Fig. 12).^344–346^ The group reported *in vitro* work in which oxidation-responsive polymersomes (PEG-*b*-PPS) were used to encapsulate and deliver the antigen and adjuvants (toll-like receptor (TLR) agonists), aimed to receptors at the endosomal compartments of DCs.^340^ The results show an increased proliferation of T cells and improved overall antigen specific immune response. Moreover, successful DC activation and maturation was detected. Later, the same group complemented this study by investigating the immune responses on priming of T cells *in vivo* with different nanocarriers.^341^ Here, either polymersomes or solid-core nanoparticles of the same oxidation-responsive block copolymer (PEG-*b*-PSS) were loaded or surface-conjugated with OVA and adjuvants.^342,343^ The authors concluded that depending on the type of nanocarrier used for antigen/agonist delivery, *i.e.* polymersome or nanoparticle, different types of T cell immune responses were enhanced (Fig. 12). For instance, while OVA encapsulating-polymersomes proved to be effective for the stimulation of antigen-specific CD4+ T cells producing inflammatory citokines, nanoparticles functionalised with OVA on their surface tended to produce a stronger CD8+ T cell response. When co-administrated, polymersomes and nanoparticles elicited immune response from both CD4+ and CD8+ T cells. This behaviour suggests that distinct antigen presentation pathways are favoured depending on the type nanocarrier in terms of physicochemical properties as well as antigen transportation method (encapsulation within a nanocarrier or displayed on the surface of a nanocarrier)*.*

As a result of the inherent pathogen-like features of vaccine delivery, polymersomes have shown promise compared to other formulations. In addition, depending on the type of carrier (polymersome, solid-core nanoparticle, micelle, filomicelle), different antigen presenting pathways or immune responses may be stimulated, which may even act synergistically and strengthen the immunogenic response. However, compared to liposome vaccine delivery systems, which were tested for Hepatitis B,^347^ Hepatitis C,^348,349^*Leishmania*,^350,351^ lymphocytic choriomeningitis virus (LCMV) glycoproteins,^352^ and widely applied in Covid-19 vaccines, polymersome-based vaccine formulations are still in an early stage of development.

### 6.2. Targeted cellular internalisation

A key step for protein delivery is, in most cases, the uptake of the polymersome into the cells. As discussed above, current formulations that are designed to mainly evade RES clearance (*e.g.*, insulin delivery), or to make use of it (*e.g.*, antigen delivery), do not necessarily need to overcome the blood circulatory system in order to be effective. However, when targeting specific tissues/organs, nanocarriers typically benefit from additional features that ease their escape from circulation and directs delivery. Blood circulation and RES clearance are therefore transient first steps that nanocarriers must undergo before being internalised into the intended tissue. As mentioned above, the fate of nanocarriers *in vivo* is strongly influenced by their properties such as size, shape, and surface functionalisation,^300,353,354^ and this also dictates their ability to escape circulation. Similarly to what is believed to occur to the discoid-shaped red blood cells,^355^ a non-spherical shape of nanocarriers influences their flow behaviour as they are prone to tumble and drift towards the endothelial wall.^300,353,354^ This facilitates their interaction with endothelial cells, and thus promotes receptor-ligand contacts that are thought to lead to faster extravasation or cell internalisation, which is especially important for active target formulations. Non-spherical polymersomes with high aspect ratio have also been shown to display a higher rate of cells adhering to the target tissues compared to spherical polymersomes.^356–359^ Among nanocarriers, polymersomes have been described to take several non-spherical shapes,^360,361^ including ellipsoidal,^356,360,362^ tubular,^360,363,364^ discoid,^365,366^ and stomatal.^366–368^

**Fig. 13:**
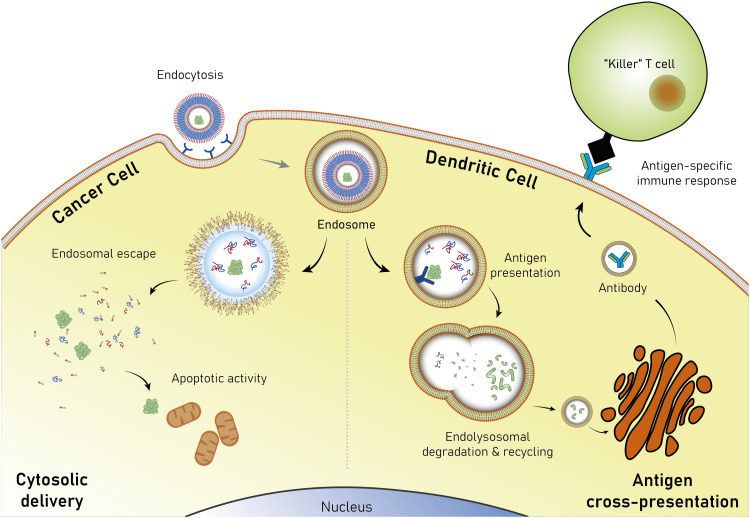
Schematic representation of two possible pathways of action in polymersome-based protein delivery. Typically, apoptotic proteins are designed for cytosolic delivery in cancer cells, where the cell machinery can be readily targeted and disrupted, once the protein successfully escapes compartmentalisation by the endosome. Conversely, immunisation may be promoted *via* antigen delivery, by targeting antigen receptors within the endosomal compartments of dendritic cells. After being broken down into fragments of interest by the endolysosomal system, the antigen is processed through the antigen cross-presentation pathway, resulting in an antigen-specific immune response.

Following extravasation from circulation, the next barriers that polymersomes encounter differ depending on the intended final target and the type of protein being delivered (Fig. 13). Previous reviews have given an in-depth discussion of the different nanocarrier strategies^37,42,369–372^ and herein we aim to highlight prominent polymersome formulations classified by which specific cells they target and which cell barriers they aim to overcome.

#### 6.2.1. Delivery to cancer cells

One of the reasons why nanocarriers are promising for cancer treatment derives from the aggressive nature of cancer growth itself, *i.e.*, the fact that endothelial dysfunction and marked blood vessel fenestrations are needed to support the abnormal tumour growth. This characteristic behaviour, although not exclusive to cancer and also observed *e.g.* upon inflammation and injury or at infection sites, along with hindered lymphatic drainage, are at the root of what is known as the enhanced permeability and retention (EPR) effect of nanoparticulate drug delivery systems, including polymersomes.^186,290^ This feature is often the rationale behind passive targeting in cancer treatments with nanocarriers, which profit from the increased permeability and preferential accumulation of the nanocarrier at the tumour site, ultimately increasing the uptake of nanocarriers into cancer cells for anti-tumour drug delivery.^373,374^ In this context, seminal work on passive delivery of protein therapeutics for cancer treatment focused on using stimuli-responsive polymersomes to trigger intracellular release of an anti-cancer model protein, cytochrome *C*, in tumour cells *in vitro*.^158^ For this purpose, a temperature- and reduction-responsive triblock copolymer composed of PEG, PAA, and poly(*N*-isopropylacrylamide) (PNIPAM; Fig. 2) was used. The resulting polymersomes were then cross-linked through amidation of the PAA blocks with cystamine, yielding temperature- and reduction-responsive vesicles. The temperature-responsiveness of the block copolymers allowed polymersome self-assembly and encapsulation of a therapeutic payload under mild conditions, whilst the disulfide-bond of the cystamine cross-linker could be cleaved in the intracellular reducing environment.

**Scheme 9:**
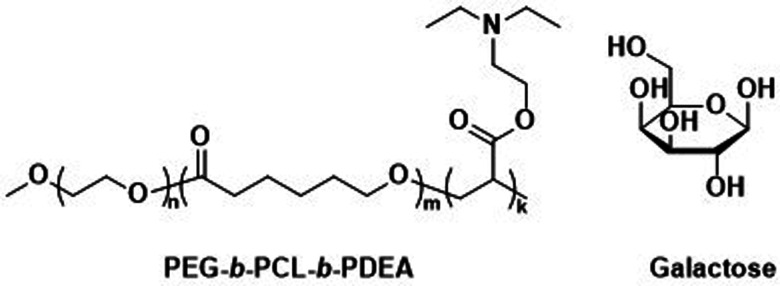
Chemical structures of PEG-*b*-PCL-*b*-PDEA used to assemble polymersomes and the structure of galactose, which has been used to decorate the surface of polymersomes to increase its binding specificity towards certain types of cancer-related receptors, such as asialoglycoprotein receptors (ASGPR).

A similar study also described the *in vitro* delivery of the same protein encapsulated with a pH and reduction-responsive PEG-*b*-PAA-*b*-poly(2-(diethyl amino)ethyl methacrylate) (PEG-*b*-PAA-*b*-PDEA) triblock copolymer (Scheme 9).^375^ In this case, the responsiveness was designed to lead to optimised cytosolic protein delivery by promoting escape from endosomal compartments, due to protonation of the PDEA block leading to the so-called “proton sponge” effect,^300^ (see Section 6.3), as well as by triggering disassembly of the polymersomes with reduction of the disulfide-cross-links within the intracellular milieu. Results from these studies using MCF-7 HeLa and 293T cell lines showed that cytochrome *C*-loaded polymersomes were taken up by the cells and that the bioactive protein was released, resulting in improved cancer cell apoptosis compared to free cytochrome *C* and control polymersomes.

**Fig. 14:**
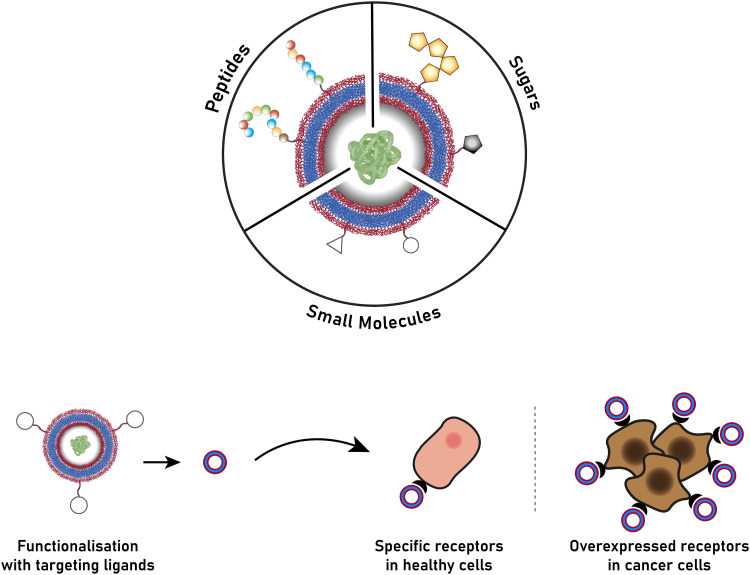
Active targeting approaches used in therapeutic protein delivery to cancer cells by polymersomes. Targeting ligands such as peptides, sugars as well as small molecules are often conjugated onto the surface of the polymer vesicles to bind to overexpressed receptors commonly found in cancer cells.

Despite encouraging performance and relative simplicity, these passive formulations were devised on the basis of an EPR effect, but the actual impact of this approach on cancer treatment remains a topic of discussion, since it both depends on and varies with the heterogeneous nature of the tumour, *i.e.* growth stage, location, microenvironment, as well as variability between individuals.^290,376–378^ Furthermore, high intratumoural pressures arising from more massive and poorly drained tumours may also limit penetration and accumulation of nanoparticles in the tumour tissue. Thus, the efficacy of nanocarrier-based formulations that solely rely on the EPR effect for tumour targeting might be compromised.^379^ Therefore, considerable efforts within the context of polymersome-based protein delivery have been made towards developing active targeted formulations.^35,290,380,381^ The most commonly encountered active-targeting polymersome formulations rely on ligands that are specific to receptors on tumours cell membranes, such as sugars, peptides, or proteins, to enhance their uptake *via* receptor-mediated endocytosis (Fig. 14).

##### 6.2.1.1. Sugar ligands

Cancer cells often overexpress surface receptors as a result of their characteristic uncontrolled growth and need for energy. Thus, sugar receptors can be potential handles for targeted delivery, if the surface of a drug-delivery vehicle is functionalised with the corresponding sugars. The most widely targeted receptors in drug delivery include lectin, mannose, galactose and hyaluronic acid receptors, as well as glucose transporters (GLUT1), and asialoglycoprotein receptors (ASGPR).^382,383^ As these receptors are typically found in hepatic cells, central nervous system (CNS) cells or dendritic cells,^383^ they constitute a good targeting handle for the corresponding types of cancer.

Galactose has a significant binding specificity to ASGPR overexpressed by hepatoma cells, which in turn facilitates internalisation by clathrin-dependent endocytosis.^384^ The first report of ASGPR-mediated polymersome-based intracellular delivery of granzyme B *in vitro*, a natural apoptotic protein, made use of reduction-responsive β-d-galactose(Gal)-decorated chimaeric polymersomes.^162^ A chimaeric character is attributed when different types of polymers constitute a multifunctional polymersome.^384^ Here, three different polymers based on PEG and PCL blocks were used: (i) PEG-*b*-PCL-*b*-PDEA, for improved loading efficiency accounting for the electrostatic interactions between the protein cargo and protonated PDEA block, (ii) PEG-SS-PCL, for de-cross-linking-induced disassembly of the polymersomes, and (iii) Gal-*b*-PEG-*b*-PCL as the ligand-bearing building block (Scheme 9). These polymersome-based artificial killer cells showed higher potency, *i.e.* lower half-maximal inhibitory concentration (IC_50_; 2.7 nM), than other anti-cancer drugs such as DOX, paclitaxel, or docetaxel. A lower apoptotic activity was observed for empty polymersomes and free granzyme B, suggesting that internalisation of granzyme B was enhanced when encapsulated in Gal-decorated polymersomes *via* receptor-mediated endocytosis.

A similar study involved the hyaluronic acid-mediated targeting of polymersomes to cancer cells. In this case, the surface of reduction-responsive chimaeric polymersomes were decorated with hyaluronic acid – a natural polysaccharide with selective binding affinity to myeloma with over-expressed CD44 receptors.^385–389^*In vitro*, these granzyme B-loaded polymersomes had a low IC_50_ of 8.1 nM and, in comparison to free DOX, proved to be at least 100-fold more potent.^390^ Moreover, further *in vivo* studies with mice bearing orthotopic human LP1 myeloma cells showed a 2.5-fold greater uptake at the tumour site than in clearing organs, such as the spleen and the liver. As a result, mice treated with these polymersomes showed the highest improvement of mean survival time compared to mice that were treated with unfunctionalised polymersomes or controls.

A mannose-receptor targeting strategy was developed to create a nanovaccine for cancer immunotherapy.^391,392^ To this end, a formulation based on mannose-decorated lipid-polymer hybrid nanocarriers for the co-delivery of ovalbumin antigen and ovalbumin receptor agonists was investigated. The system aimed at activating dendritic cells and triggering an enhanced immune response in tumour-bearing mice through antigen-specific cytotoxic T cells. *In vitro* studies showed improved cellular uptake of encapsulated antigen compared to free antigen-antagonist combinations. Moreover, vesicular delivery resulted in the highest levels of dendritic cell maturation, which is necessary for an effective immunogenic response. *In vivo*, an increased antigen depot was noted for mannose-functionalised nanocarriers as well as an upregulation of antigen-specific T cells. As a result, the functionalised lipid-polymer hybrid vesicles were two-fold better at inducing the production of apoptotic granzyme B and specific cytokines compared to their unfunctionalised analogues. Moreover, such mannose-decorated vesicles were the most effective in retarding tumour progression with some tumour free cases up until the end of the experiment (192 days), highlighting the long-term immune response achieved.

These sugar-receptor based formulations display perhaps the broadest therapeutic options, as encouraging results were found for either direct delivery of apoptotic proteins, as well as for long-term vaccines for antigen delivery. *In vivo* studies have already been performed with other mannose-functionalised formulations, such as liposomes^393,394^ and dendrimers,^395^ which have also displayed robust immunological responses. An area that has not been explored using sugar-functionalised polymersomes is anticancer protein delivery to brain cells, where liposomal^396^ or nanoparticle^397^ formulations have been previously employed for glioma therapies. Nevertheless, since the antitumor performance of such polymersomes levels with several other equivalent formulations, it can be expected that more studies of sugar-mediated polymersomes therapies will soon follow.

##### 6.2.1.2. Peptide ligands

The use of peptides in targeted drug delivery has been gaining increasing momentum in comparison to their high-molecular-weight counterparts, such as antibodies, enzymes or proteins. Particularly, their lower cost, lower susceptibility to either chemical or biological degradation, lower conjugation complexity, and lower degree of immunogenicity,^309,398^ make peptides more convenient to use, while a performance similar to that of other protein targeting ligands is attained. Consequently, polymersomes decorated with either peptide motifs that target proteins or with cell penetrating peptides are the most common strategies in protein delivery formulations for cancer therapies.

Cell growth *in vivo* greatly relies on a matrix support, which is established through anchoring of the cells *via* adhesion molecules, such as integrins, to fibres of the extracellular matrix. Conversely, with uncontrolled proliferation and invasiveness of cancer cells, those integrins are overexpressed to support tumour growth, which makes such surface receptors attractive for cancer-targeting purposes. While several types of integrins and constituting subunits exist, even within the same cell, some are usually associated with cancer-related processes, such as migration, proliferation, or blood vessel formation. Currently, the few reports of polymersome-based formulations delivering therapeutic proteins that take advantage of integrins are based on peptides containing the Arg-Gly-Asp (RGD) sequence. For instance, a PEG-*b*-PBD polymersome (Scheme 5) decorated with a biomimetic targeting peptide, PR_b, (sequence: KSSPHSRN(SG)5RGDSP) has been described for the delivery of the apoptosis-inducing protein tumour necrosis factor alpha (TNF-α) to human prostate cancer cells (LNCaP).^399^ This PR_b peptide resembles the cell adhesion binding site and also features a specific amino acid sequence which, as a synergistic secondary binding site to integrins, allows to better mimic the cell surface-matrix adhesion. *In vitro* tests showed a successful binding to the surface of LNCaP cells and transportation of the functionalised polymersomes into the intracellular space, with the amount of internalisation being proportional to the degree of the polymersome's surface functionalisation with PR_b. Loaded with TNF-α, these polymersomes were found to be four-fold more cytotoxic to the prostate cancer cells than free TNF-α and non-functionalised polymersomes. Conversely, comparisons to a previous *in vitro* study performed by the same group with PR_B-functionalised PEGylated liposomes^400^ indicated that the difference between unfunctionalised and PR_b-functionalised vesicles is two-fold more accentuated with polymersomes than with liposomes in cell cytotoxicity tests with LNCaP cells. The authors argued that this difference arises most likely from the fact that unfunctionalised polymersomes were less prone to non-specific interactions with the targeted cells than their liposome counterparts, due to a higher PEG surface coverage of the polymersomes. Nevertheless, the liposomes resulted in an overall increased cell cytotoxicity compared to the polymersomes which the authors attributed to the intrinsic leakiness of liposomes and, therefore, a better cargo release from the liposomes within those cells.

**Scheme 10:**
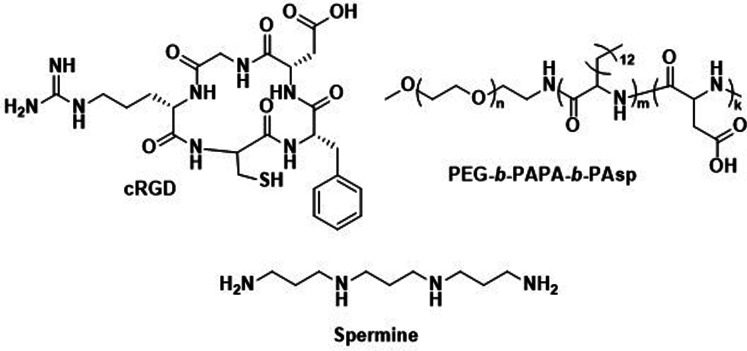
Chemical structures of cRGD, a cyclic peptide specific to receptors in lung tumours, PEG-*b*-PAPA-*b*-PAsp block copolymer, used to make polymersomes, and spermine, used to graft onto the polymersome to improve protein loading efficiency.

In another study, a cyclic RGD peptide (cRGD; Scheme 10) was used to specifically target lung tumour cells in mice which overexpressed α_v_β_3_ integrin.^401^ A chimaeric polymersome, constructed from a PEG-*b*-poly(α-aminopalmitic acid)-*b*-poly(l-aspartic acid) (PEG-*b*-PAPA-*b*-PAsp) and cRGD-PEG-*b*-PAPA (Scheme 10), was used to encapsulate and deliver saporin, a ribosome inactivating protein used in clinical trials for the treatment of leukaemia and lymphoma.^402^*In vitro* studies showed a two-fold greater internalisation of functionalised polymersomes compared to the unfunctionalised ones.^401^ With an IC_50_ of 16.3 nM, the functionalised polymersomes showed nearly twice as much cell death potency than unfunctionalised polymersomes (29.2 nM). It should be noted that free saporin exhibited very low potency, as a result of poor uptake. *In vivo* studies highlighted, however, that the functionalised and non-functionalised polymersomes performed very similarly in terms of accumulation of model protein cytochrome *C* within the tumour. Likewise, when delivering anti-tumour saporin, both functionalised and unfunctionalised polymersomes resulted in similar survival rates of the treated mice, with functionalised polymersomes performing better, regarding the retardation of tumour growth. Both these examples highlight the need for well-designed control and comparison experiments in order to quantitatively determine the advantages of targeted polymersome-based formulations compared to non-functionalised polymersomes and liposomal analogues.

To further improve the release of proteins from the polymersome once internalised, a more complex cRGD-decorated chimaeric polymersome formulation was developed that targets α_ν_β_3_-positive A549 lung cancer cells.^403^ The polymersomes were based on PEG-*b*-P(TMC-*co*-DTC) block copolymers (Scheme 6), covalently bound to either (i) cRGD, (ii) polycationic spermine (Scheme 10) for the optimisation of the loading efficiency, or (iii) maleimide to enable cleavable cross-linking for increased leakiness in reducing environments in cells. Moreover, incorporation of the pH-triggered fusogenic peptide GALA (sequence: WEAALAEALAEALAEHLAEALAEALEALAA) on the surface of the polymersome facilitated the escape of the cargo from late endosomes (discussed below). A mix of these four differently functionalised polymers was used for the self-assembly of polymersomes. *In vitro* studies showed a similar targeting ability of GALA-cRGD and polymersomes decorated solely with cRGD. A greater potency in terms of anti-cancer activity and cell apoptosis was observed for the GALA functionalised polymersomes, registering a minimum IC_50_ of 5.7 μM.

**Fig. 15:**
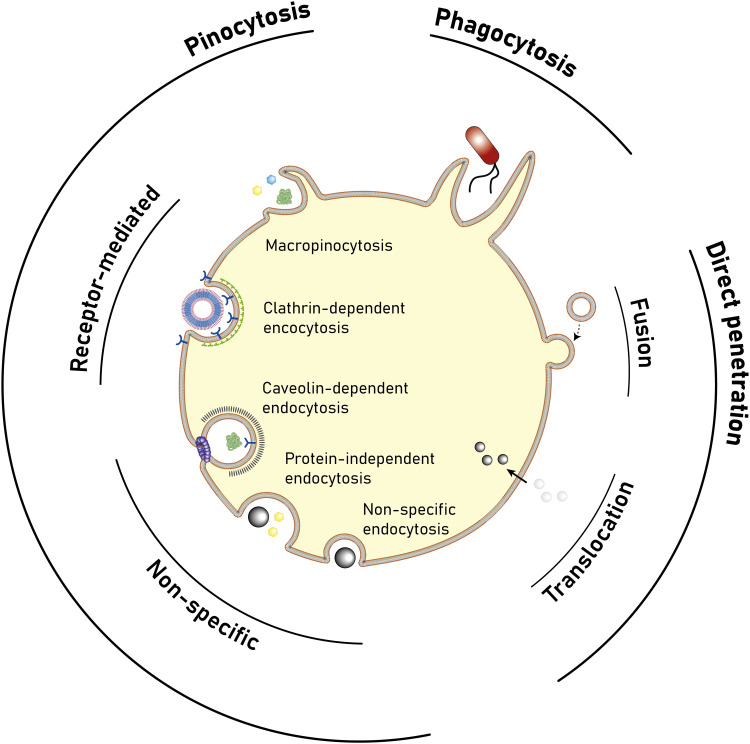
Schematic representation of common cellular uptake pathways. The specific uptake route is dictated by factors such as the specific environment and type of the cell, as well as the physico-chemical features of the uptaken entity. For instance, cell penetrating peptides may be internalised *via* endocytosis, whether by means of receptor-mediated (aided by scaffold proteins such as clathrin or caveolin) endocytosis or non-specific (protein-independent) endocytosis. However, they are also known to be able to enter the cell by directly penetrating through the membrane (translocation).

Another therapeutic formulation approach is the use of cell-penetrating peptides (CPPs).^404,405^ These are short sequences of amino acids, usually bearing a positive charge at physiological pH, and are known to penetrate cellular membranes. The mechanisms for cellular internalisation of CPPs are not yet fully understood, but CPPs can undergo internalisation either by endocytic pathways, *i.e.* macropinocytosis, clathrin-mediated endocytosis, and caveolae/lipid raft-mediated endocytosis, or by non-endocytic pathways where the peptide can directly penetrate the cell membrane (Fig. 15).^404,406^ The specific pathways or combination thereof depend on factors such as the type of CPP and targeted cells, environmental parameters (*e.g.* temperature, pH), and concentration. Nevertheless, it is believed that endocytosis happens in most cases, while higher concentrations may favour a direct penetration of CPPs.^405,407,408^

**Scheme 11:**
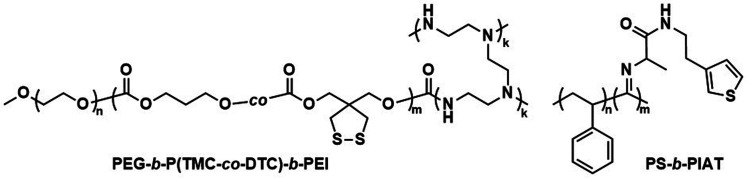
Chemical structures of PEG-*b*-P(TMC-*co*-DTC)-*b*-PEI and PS-*b*-PIAT block copolymers used to make polymersomes for targeted delivery using peptides as ligands.

Currently, only one *in vitro* and one *in vivo* study in which CPP-functionalised polymersomes were used have been reported. In one of them, polymersomes based on polystyrene-*b*-poly[l-isocyanoalanine(2-thiophen-3-yl-ethyl)amide] (PS-*b*-PIAT; Scheme 11) were investigated.^409^ This polymer was conjugated with an arginine-rich HIV-1 *trans*-activator of a transcription (TAT) protein (sequence: GGGGYGRKKRRQRRR), as means to facilitate penetration of the polymersome into the cell for delivery of model proteins, such as green fluorescent protein (GFP) and horse radish peroxidase (HRP).^409^ The *in vitro* results indicate an improved cellular internalisation of TAT-functionalised polymersomes *via* macropinocytosis (Fig. 15). In the other example, chimaeric polymersomes based on PEG-*b*-poly(trimethylene carbonate-*co*-dithiolane trimethylene carbonate)-*b*-polyethylenimine (PEG-*b*-P(TMC-*co*-DTC)-*b*-PEI; Scheme 11) containing reversible cross-links were decorated with a CPP33 protein (sequence: RLWMRWYSPRTRAYG) for the intracellular delivery of granzyme B and cytochrome *C* into orthotropic A549 human lung tumour xenografts.^410^ Incubation with A549 cells with functionalised polymersomes showed a two-fold increase in accumulation of fluorescently-labelled cytochrome *C*, compared to unfunctionalised polymersomes, and an eleven-fold increase compared to free cytochrome *C*. These studies suggest that either direct penetration or receptor-mediated endocytosis lead to internalisation of CPP33-functionalised polymersomes. Conversely, polymersomes encapsulating granzyme B, when incubated in the same cells, feature half (20.7 nM) of the untargeted polymersomes’ IC_50_. On the other hand, anti-tumour activity assessments with CPP33-functionalised polymersomes lacking granzyme B clearly demonstrated that the observed cell death was mainly due to the apoptotic activity of granzyme B and not a direct consequence of the cytotoxic effects of the CPP. *In vivo* treatment of mice bearing orthotopic A549 lung tumour cells showed complete tumour growth inhibition, and the survival time of the mice was doubled compared to control experiments.

The above studies emphasise the promise of using peptides as targeting and cell penetrating moieties in formulations for anticancer therapies. Nevertheless, the main issues that CPPs face are that their benefits in anticancer therapeutics over established methods are yet to be clearly demonstrated,^411,412^ and that a proper understanding of their functioning and cell uptake pathways is still missing. An alternative approach, which has not been explored for protein delivery using polymersomes, is the use of antibodies as targeting moieties, as such systems could capitalise on the unmatched specificity of antibodies. In addition, antibody-based targeting of tumour cells has been successfully demonstrated for other types of nanocarriers.^413,414^

##### 6.2.1.3. Small molecule ligands

**Scheme 12:**
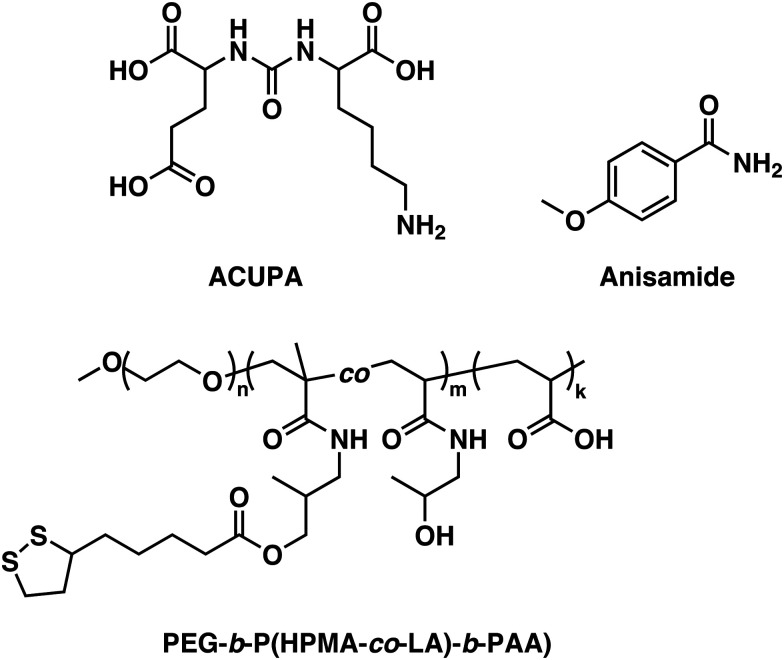
Chemical structures of 2-(3-[5-amino-1-carboxypentyl)-ureido]-pentanedioic acid (ACUPA) and anisamide employed as cancer-targeting ligands on polymersomes for delivery of therapeutic proteins, as well as PEG-*b*-P(HPMA-*co*-LA)-*b*-PAA) block copolymer used to assemble polymersomes for targeted delivery.

In contrast to the more complex and delicate targeting ligands mentioned above, small organic molecules present themselves as straightforward alternatives to enhance the uptake of polymersomes into cells. Their main advantages are their availability, low cost, and the relative ease with which they can be attached to polymersomes compared to their more sensitive biomacromolecular counterparts. Particularly, two molecules have been extensively studied in cancer therapeutics, namely 2-[3-(5-amino-1-carboxypentyl)-ureido]-pentanedioic acid (ACUPA) and anisamide (Scheme 12).

ACUPA is a widely investigated small organic molecules that is used to target prostate-specific membrane antigen (PSMA) receptors, which are commonly overexpressed in prostate cancer cells,^414–417^ while anisamide is a benzamide derivative that is thought to selectively target the family of Sigma receptors overexpressed in cancer cells.^418–420^ Polymersomes constructed from PEG-*b*-poly(2,4,6-trimethoxybenzylidene-pentaerythritol carbonate)-*b*-poly(succinic acid carbonate) (PEG-*b*-PTMBPEC-*b*-PSAC), with pH-cleavable acetal moieties in the PTMBPEC block, were functionalised through chemical conjugation with ACUPA to deliver granzyme B to prostate cancer cells.^421^ The *in vitro* studies indicated that this formulation had a significant antitumor activity with an IC_50_ of 1.6 nM which was ascribed to the effective targeting and internalisation of the polymersome. It was also observed that the cell death potency was dependent on the content of ACUPA on the polymersomes, with functionalisation degrees below 10 wt% resulting in increased cytotoxicity while no further improvement was registered at higher degrees of functionalisation. *In vivo* studies for the ACUPA receptor strategy have not been yet reported.

Similar pH-responsive polymersomes were decorated with anisamide *via* chemical conjugation. These constructs allowed delivering granzyme B to non-small cell lung cancer (NSCLC) cells, and a low IC_50_ of 3.75 nM was determined. A self-cross-linked reduction-responsive polymersome formulation based on PEG-*b*-poly(*N*-2-hydroxypropyl methacrylamide-*g*-lipoic acid)-*b*-poly(acrylic acid) (PEG-*b*-P(HPMA-*co*-LA)-*b*-PAA; Scheme 12), functionalised with anisamide and encapsulating granzyme B, was tested *in vivo* with mice bearing human lung xenografts.^422^ The functionalised polymersomes were observed to deliver the model protein cytochrome *C* more efficiently into the tumour cells compared to non-functionalised polymersomes. Formulations encapsulating granzyme B showed a clear tumour growth inhibition and improved median survival rates of the mice, compared to non-functionalised polymersomes. This was attributed to the lack of targeting capabilities of the latter and, therefore, a more pronounced tumour penetration of the anisamide-functionalised polymersomes. Both these examples highlight the promise of small organic molecules to achieve targeted protein delivery with polymersomes. While molecules such as ACUPA have been extensively described to be promising selective targeting ligand,^417^ more complete *in vivo* studies are warranted to consolidate its value in the targeted delivery of polymersomes. Similarly, the use of anisamide has resulted in promising results. However, in contrast to ACUPA, the selective targeting mechanisms of anisamide is still to be fully understood. In fact, among several anisamide-decorated formulations, there are conflicting reports regarding their cell specificity and, therefore, the targeting capabilities of anisamide.^423^ While many of the reported studies show promising outcomes, careful thought must go into avoiding lingering issues like unspecific interactions with off-target cells. Factors influencing such events are commonly negligible within *in vitro* investigations, thus more extensive research *in vivo* might establish a better control over cellular uptake of such formulations.

#### 6.2.2. Blood–brain barrier

**Fig. 16:**
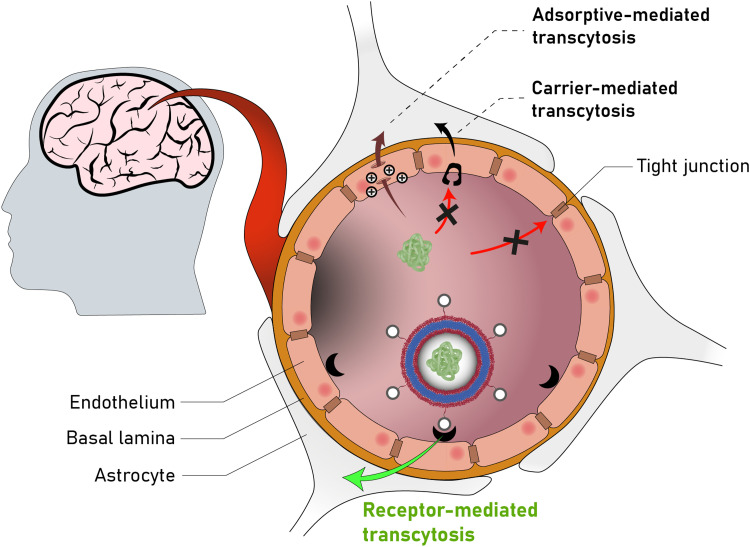
Schematic representation of specific and non-specific pathways for transport across the blood–brain barrier (BBB) into brain cells (astrocytes). The tight junctions between the cells forming the endothelium are at the base of the BBB‘s restrictive nature. Non-specific pathways include adsorptive-mediated transcytosis (AMT) and carrier-mediated transcytosis (CMT), typically associated with the transport of cationic proteins and nutrient small molecules (*e.g.* ions, sugars and amino acids), respectively. Specific pathways such as receptor-mediated transcytosis (RMT) rely on specific interactions with receptors that typically populate the endothelium, such as insulin or transferrin receptors, which are commonly used to promote protein delivery with polymersomes.

While the central nervous system's control of the body's functions heavily relies on traffic of substrates in and out of cells, the blood vessels that feed the brain are specialised in regulating the transport of molecules, thereby protecting the brain from pathogens or toxins, and assuring its proper functioning. This blood–brain barrier (BBB) is remarkably impermeable because of a continuous epithelium with tight junctions, which prevents transport of molecules between cells. Nevertheless, the brain still has high maintenance requirements in terms of nutrients and all sorts of regulatory endogenous metabolites. Transcytosis, *i.e.*, the crossing of the barrier, is limited to a small set of molecules and this limits the effect of most therapeutics for brain diseases. The main pathways of mass transport across the BBB include transcellular passive diffusion, carrier-mediated transport, receptor-mediated transcytosis, and adsorptive transcytosis (Fig. 16).^424,425^ Among these mechanisms, receptor-mediated transcytosis is particularly attractive for the delivery of therapeutics, including proteins and nanocarriers, as several transcytosis related receptors are highly expressed at the brain endothelium, including transferrin receptors,^426^ the low-density lipoprotein receptor family,^427^ and others.^428–431^ The use of these receptors has thus become a strategy to overcome the BBB for the delivery of therapeutics, especially proteins (Fig. 16).^425,428^

Some receptors, such as transferrin and lactoferrin receptors, are known as suitable gateways for crossing the BBB. In the context of protein delivery, in a pioneering study, polymersomes constructed from PEG-*b*-PLGA were decorated with mouse-anti-rat monoclonal antibody OX26, a type of transferrin receptor antibody, for delivery in mice.^432^ A model peptide, NC-1900, was encapsulated. The formulation led to an accumulation of this peptide in the brain. It should be noted that a translation to human therapies may be challenging as the OX26 ligand is only present in rats.^433^ In another study, lactoferrin-functionalised polymersomes were investigated for the delivery of an anti-apoptotic, neuroprotective agent, S14G-humanin, in mice.^434^ The *in vivo* tests showed a two-fold improvement in brain tissue accumulation compared to non-functionalised polymersome formulations and showed an attenuation of disease-induced brain deterioration. An increase in the surface density of lactoferrin on the polymersome led to higher accumulation and faster plasma clearance with a limit after which saturation was detected. However, a three-fold increase in off-target accumulation of the functionalised polymersomes was registered in the liver and lungs compared to non-functionalised formulations, putting the specificity of this receptor for the brain into question.

The currently more robust strategies for delivery across the BBB rely on targeting other well-characterised BBB receptors, such as low-density lipoprotein receptor (LRP), by decorating the polymersome surface with either Angiopep-2 or alipoprotein E.^430^ Angiopep-2 is an oligopeptide featuring an affinity to low-density lipoprotein receptor-1 (LRP1) and is currently involved in non-polymersome formulations undergoing clinical trial for the treatment of brain cancer.^435,436^ Given the promising potential of Angiopep-2, investigations have focused on the effectiveness of this ligand for the targeted delivery of saporin to xenografted glioblastoma – an aggressive and high-mortality malignancy – in nude mice.^166^ Self-cross-linked reduction-responsive chimaeric polymersomes, based on PEG-*b*-P(TMC-*co*-DTC)-*b*-PEI (Scheme 11), were functionalised with Angiopep-2 for preliminary *in vitro* studies. The results showed a low IC_50_ of 30.2 nM and a two-fold improvement in internalisation compared to unfunctionalised polymersomes. This led to a significant potency against cell growth while free saporin had negligible inhibitory effects. *In vivo* investigations in glioblastoma-bearing nude mice showed that the polymersomes could cross the BBB as well as accumulate at the target site. Optimal targeting was observed to closely relate to the surface density of the ligand on the polymersome as crowding led to non-specific interactions, and ultimately hindered accumulation at the target site. The Angiopep-2 receptor formulation showed a four to seven-fold decrease in tumour growth and improved median survival time. However, relatively high levels of non-specific accumulation in the liver, kidneys, and spleen were detected even though no major cytotoxic effects were observed.

A key investigation regarding the BBB crossing mechanism of Angiopep-2-functionalised pH-responsive poly(2-(diisopropylamino)ethyl methacrylate) (PDPA)-based polymersomes was performed by Battaglia and colleagues.^437^ The results of an *in vitro* study that involved mouse brain endothelial cells and astrocytes suggested that transcytosis was indeed strongly manifested for Angiopep-2 functionalised polymersomes. In addition, it was observed that LPR-1 transcytosis occurred through a non-acidifying pathway, bypassing endosome/lysosome acidification, as pH-responsive polymersomes were unaffected during the process. Further *in vivo* studies in mice found that functionalised polymersomes were located in deeper regions of the brain compared to the unfunctionalised version, showing a clear reach to deeper regions of the brain and spinal cord. Interestingly, the results also suggest some sort of clearance mechanism for these nanocarriers to be present in the brain and spinal cord as their detection vanished faster than their average plasma residence time. Moreover, also the elasticity of polymersomes influences the crossing of the BBB, as demonstrated with Angiopep-functionalised polymersomes.^438^ Rigid, more crosslinked polymersomes accumulated in higher concentration in an orthotopic glioblastoma tumor model than more flexible and softer polymersomes.

In another study, alipoprotein E-functionalised PEG-*b*-PLA polymersomes were loaded with β-galactosidase (β-gal) to treat GM1 gangliosidosis – a genetic lipid storage disorder of the brain and spinal cord, *in vitro*.^439^ Results from this enzyme-based therapy show that the β-gal activity was restored to normality, and was 22-fold higher than free β-gal. However, unfunctionalised polymersomes had similar levels of β-gal activity which was attributed to a lack of upregulated LRP receptors in the cells and to simultaneous non-specific uptake pathways by endocytosis.

The above examples show that both Angiopep-2 or alipoprotein E constitute valid options to enhance the BBB crossing of polymersomes. Only one study currently exists in which these two receptors are compared in terms of efficiency for polymersome-based protein delivery therapies.^80^ Using an *in vitro* BBB model, alipoprotein E functionalised polymersomes showed a 2.2-fold better crossing of this barrier for immortalised mouse brain endothelial cells compared to Angiopep-2 functionalised ones. Both *in vitro* and *in vivo* data show that the alipoprotein functionalised polymersomes led to better transcytosis, higher accumulation in the tumour cells, lower IC_50_ values, and better survival rates for mice compared to the Angiopep-2 analogue. This was thought to arise from the multiple receptors that alipoprotein E can target, namely LPR-1, LPR-2, and low density lipoprotein receptor (LDLR),^440^ compared to the single-receptor targeting Angipep-2.

When considering the delivery of therapeutic proteins across the BBB, polymersomes are still at early stages compared to polymersome-based targeted delivery to other cells discussed previously. This is in part the result of the restrictive permeability and complexity of the BBB, which constitutes an obstacle common to all available drug delivery vehicles. Evidence of this is the typically low protein/therapeutic cargo accumulation, and therefore efficacy, featured by such formulations. Conversely, the main strategy to overcome the BBB is through receptor-mediated internalisation, with the difficulty of choosing a receptor unique to the BBB, which is, *e.g.*, not the case for the above-mentioned transferrin. Driven by this issue, the focus has been directed towards more brain-selective receptors, such as LDLR. However, even with targeting ligands such as alipoprotein E and Angiopep-2, insufficient levels of predictability of polymersome formulations with respect to their mechanism of cellular uptake hinder the advancement into clinical trials. Regarding other strategies to overcome the BBB for protein formulations, a wide range of nanocarrier formulations have been investigated, including liposomes,^441–444^ nanoparticles,^445–450^ micelles,^451,452^ dendrimers,^453^ and drug-protein conjugates,^454^ using the aforementioned ligands as well as other active targeting functionalities,^425,443,455^ with some of these currently undergoing clinical trials.^425,455,456^ Considering the diversity of promising BBB-specific targeting moieties available, the same extent of research has still to be met with polymersome formulations.

### 6.3. Endosomal escape of therapeutic proteins from polymersomes

In most cases, cellular uptake of polymersome formulations relies on their compartmentalisation into endosomes, which are not the final destination of the cargo but rather an obstacle to overcome (Fig. 13).^457^ Endosomal escape of nanocarrier cargo is, therefore, of paramount importance for the development of safe and effective formulations, especially for intracellular delivery of bioactive proteins, which are particularly susceptible to degradation within the endosome.^458^ Yet, relatively little is known about this process.^459^ As reflected in many of the studies discussed in this review, it is common practice to demonstrate that the polymersome cargo is delivered intracellularly by means of fluorescent cargo or co-localisation analysis *in vitro*, or through *ex vivo* analysis. Endosomal escape is often implied from such analyses. However, few studies attempted to address the underlying mechanism for endosomal escape, as it usually does not constitute the central question in the reported work on polymersome-based formulation for protein delivery, and no systematic investigations have been reported.

**Fig. 17:**
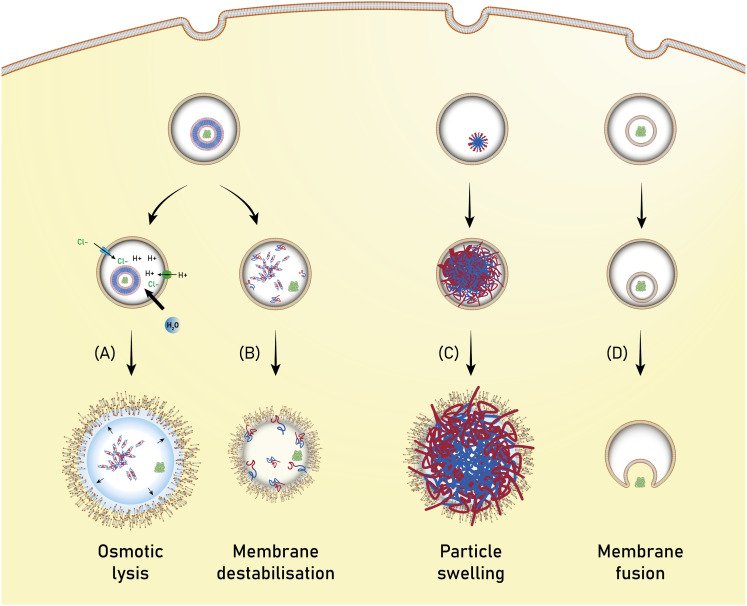
Schematic representation of proposed endosomal escape mechanisms of nanocarriers such as polymersomes, liposomes and nanoparticles. (A) Osmotic membrane rupture *via* the so-called “proton sponge” effect where the buffering capacity of pH-responsive polymersomes (or other polymeric nanocarriers) causes an influx of counter ions leading to an increase in osmotic pressure, ultimately causing endosome/lysosome membrane rupture. (B) Disassembly of pH/reduction-responsive polymersome into amphiphilic unimers that destabilise the endosome/lysosome membrane. (C) Swelling of pH-responsive nanocarriers (*e.g.* nanoparticles) inducing mechanical stress over the membrane. The swelling may also be associated with osmotic rupture/”proton sponge” effect. (D) Fusion of the nanocarrier, typically liposomes, with the endosome/lysosome membrane. (A and B) are the mechanisms most commonly associated with polymersome-based protein delivery.

Generally, the endosomal escape of polymersome-based delivery formulations occurs *via* osmotic rupture or membrane destabilisation (Fig. 17A and B, respectively),^457–459^ however an interplay between both, or other uncharacterised mechanisms, cannot be ruled out. The osmotic rupture is often associated with the so-called “proton sponge” effect, which is the most commonly attributed mechanism to cause endosomal escape.^186,290^ This is often the rationale for including at least one pH-responsive polymer block into the block copolymers that form the polymersomes, typically through selection of amine-bearing moieties. The “proton sponge” effect is postulated to rely on the buffering capacity of the pH-responsive polymer which, upon endosomal acidification, sequesters surrounding protons.^186,290^ Such event is accompanied by an influx of counter ions, causing an increase in osmotic pressure that eventually induces destabilisation and further rupture of the endosome membrane. It should be noted that, despite the “proton sponge” effect being frequently attributed to facilitate endosomal escape, there is growing debate over the actual impact of the buffering capacity underlying the main mechanism.^460,461^ Nevertheless, the “proton sponge” effect is commonly argued to be the underlying mechanism triggering endosomal escape involving pH-responsive polymersomes.^155,375,462^ An alternative perspective of the typical “proton sponge” effect suggests the triggered disassembly of nanocarriers into unimers (*i.e.* individual polymer molecules) as the driving force for increased osmotic pressure causing endosome lysis.^463^ A nuanced version of this osmotic mechanism was also described, where a light-sensitive photosensitiser disrupted the hydrophobic-hydrophilic balance of the block copolymers.^464^ The PEG-*b*-PPS based polymersomes contained a photosensitiser, ethyl eosin, which oxidatively increased the hydrophilic character of the PPS block during short illumination with near-UV light. This caused a reassembly of the polymersomes into smaller micelles, leading to an increase in osmotic pressure. A faster and more pronounced lysis was, however, attributed to the swelling of the endosomes arising from the osmotic influx of reactive oxygen species, as well as temperature increases from light absorption. This culminated in the destabilisation of the endosomal membrane and a consequent escape of the ovalbumin antigen cargo into the cytosol.

Conversely, other reported forms of endosomal escape rely on arginine-rich cell penetrating peptides, like TAT, which are known to promote cellular uptake because their positively charged character leads to non-specific interactions with the negatively-charged cellular membranes.^410,465–467^ However, there is still an ongoing discussion whether these types of cell penetrating peptides are in fact responsible for facilitating endosomal escape,^466^ with a pertinent example being the work of van Hest's group.^409^ Their *in vitro* study using TAT decorated-polymersomes assessed the endosomal escape of encapsulated GFP. The results indicate that cargo was found entrapped in the acidic endosomal compartments but also in other regions, which could not be distinguished between non-acidic compartments and/or cytosol.^410,465–467^ Consequently, a clear relation between TAT and putative escape events could not be established, thus highlighting the question around the functioning of the mechanism of TAT-induced endosomal escape. In another study, cytosolic delivery of an apotoptic protein (granzyme B) with CPP-functionalised polymersomes was achieved.^410^ The fast uptake was attributed to the CPP, namely CPP33, which was hypothesised to promote both cellular uptake and further endosomal escape. Other types of cell penetrating peptides thought to disrupt or destabilise endosomal membranes are fusogenic: pH-responsive oligopeptides able to mediate membrane fusion with endosomal membranes, as verified for some viruses, liposomes, and lipid/polymer hybrid polymersomes. The use of such fusogenic cell-penetrating peptides to facilitate endosomal escape was also been demonstrated with polymersomes whose surface was functionalised with the fusogenic peptide GALA.^403^ These were used for the *in vitro* delivery of apoptotic cytochrome *C* to A549 human lung cancer cells and showed effective delivery into the cells, while their non-functionalised counterparts were contained mainly inside endosomal compartments. Although the actual mechanism of endosomal escape was neither further assessed nor associated with a possible membrane fusion event, the conformational change of GALA from a random coil to an α-helix at different pH values was thought to cause membrane destabilisation and consequent endosomal escape.

Besides osmotic pressure and membrane destabilisation reported in the few studies of polymersome-based protein delivery that address endosomal escape, other mechanisms have been described to induce endosomal escape in other formulations, including nanoparticle swelling and membrane fusion.^468–470^ The former is characterised by the swelling of pH-responsive nanoparticles triggered by the acidic milieu of endosomes/lysosomes. However, it is still under debate whether escape from the endosomes is facilitated by the mechanical stress caused by the nanoparticles’ swelling or if it is again a consequence of the osmotic pressure arising from a “proton sponge” effect.^471,472^ Membrane fusion, on the other hand, relies on coalescence of the nanocarrier with the endosomal membrane to allow the cargo to escape to the cytosol. Though mostly described in lipid-based nanocarriers due to the similarity of liposomal membranes with endosomal membranes, membrane fusion has also been reported with polymer formulations.^473,474^ It is noteworthy to mention that such endosomal escape mechanisms are still far from being consensual, as they are yet to be fully characterised and because conflicting results can be found in the literature. Nevertheless, the polymersome field could benefit from exploiting these mechanisms in order to optimise and diversify polymersome-based formulations and, perhaps, further elucidate the questions revolving around endosomal escape.

In a different framework, some formulations can actually benefit from protein release within the endosomal compartments, without the need for the protein to reach the cytosol to perform its function (Fig. 13). This is commonly the case for antigen/adjuvant delivery to certain subtypes of dendritic cells which display receptors, *e.g.* Toll-like receptors, that allow for antigen cross-presentation, and thus enhance antigen-specific immune responses.^344^ Indeed, two stimuli-responsive polymersome-based formulations for ovalbumin antigen delivery indicated such endosomal presentation pathways in *in vitro* studies (Section 6.1).^340,391^ It is noteworthy to underline that, in these studies, the efficacy of the immune response was also argued to be favoured through cytosolic delivery of the antigens in addition to the endosomal presentation pathway.

Overall, the examples discussed throughout this section are intended to highlight the complex nature of endosomal escape and the limited knowledge available in the scientific literature on this late, but crucial, stage of the polymersome-based protein delivery journey. This is an issue not unique to polymersomes but for all types of nanocarrier formulations and highlights the lack of fundamental knowledge of this aspect. Comparative studies between different nanocarriers, which may aid in the elucidation of these mechanisms, have also not been reported to date. Nonetheless, there seems to be a general acceptance that endosomal escape of polymersomes does in fact occur, as outlined with the examples addressed herein, even if through as-of-yet not fully understood or characterised mechanisms. However, the often-implied undefined endosomal escape events that precede successful protein cargo delivery may limit more in-depth assessments of such relationship.

### 6.4. Cellular uptake as a barrier to clinical translation

In the past few decades a great deal of effort has been directed towards the development of polymersome-based formulations that successfully deliver therapeutic proteins. Escaping the natural clearance mechanism, achieving cellular uptake despite various barriers, and the escape of the protein from the endosome are the main factors determining the effectiveness of such formulations. While a significant amount of progress and diversity can be found in the literature, a major challenge is the modular manner in which these biological barriers are tackled, most likely a result of the relatively recent beginning of polymersome-based protein delivery research. Yet, despite an abundance of *in vitro* studies that highlight the potential of polymersomes bearing targeting moieties to encapsulate and release bioactive proteins in cell lines, their translation into *in vivo* studies – where biological barriers play a much more significant and intricate role – are scarce. Moreover, the evaluation of existing *in vitro* studies of polymersome formulations is not straightforward as each specific experimental setup, cell line, or protein cargo may yield varying results for the same formulation, which oftentimes limits an accurate assessment of the efficacy of the underlying cellular uptake approach used. Overall, direct comparative studies or controls against other equivalent formulations, such as liposomes, in the same experimental setups would remove limitations of the current case-by-case evaluation of their effectiveness and provide a clear frame of reference with respect to formulations in clinical trial or already on the market.

Particularly the intrinsic complexity of polymersome-based formulations, with respect to targeting ligands, choice of block copolymers, *etc.*, represents a considerable disadvantage in comparison with other, often simpler therapeutic formulations that are more advanced in the clinical translation process, such as liposomes^35^ or protein-nanoparticle conjugates.^309^ In fact, the efficacy of a given formulation is just one of the many factors contributing to a successful clinical translation.^35^ It also requires cost-effective, quick, and scalable production as well as straightforward quality control. Moreover, increased complexity or diversity of entities present in a formulation also raises concerns on safety issues and accumulation of potential failure-points, such as unpredictable non-specific interactions, accumulation of nanocarriers at undesired sites in the body, and detrimental side-effects. In this regard, current polymersome-based vaccine or insulin delivery approaches might face difficulties not necessarily because of insufficient performance, but because of the use of synthetic polymers that raise questions about potential system toxicity. This is less of an issue with liposomes or lipid nanoparticles that are based on naturally occurring lipids. In addition, polymersomes also have to compete with conventional vaccination, cancer or diabetes treatments that underwent strict clinical trials and are, therefore, already in place.

## 7. Conclusions and outlook

Since their discovery in the late 1990s, polymersomes have been hailed as promising nanocarriers for drugs and proteins for a variety of therapeutic applications. Particularly their thick membrane and their ability to encapsulate both hydrophilic and hydrophobic molecules constitute clear advantages over other polymer-based nanocarriers such as micelles. Furthermore, the stability of polymersome membranes at ambient temperature, originating from the inability of the long polymer chains to leave the self-assembled structure, improve their shelf-life compared to liposomes. Limitless ways to modify the polymer backbone provide access to a virtually infinite number of polymersomes to be generated, which is not the case for other types of nanocarriers, whose design is limited by fewer available building blocks, such as lipids. Nonetheless, the translation of polymersomes into clinically relevant therapies has not yet been achieved. While the comparatively short time since their discovery certainly plays a role, other aspects such as biodegradation of polymersomes, manufacturing methods, and the intracellular fate also contribute to it. In all these aspects, liposomal protein delivery methods have defined advantages that are highlighted by the fact that they are already on the market and in numerous clinical trials. Thus, the polymersome field may be well served to learn from the more advanced liposome field and adapt methods, such as manufacturing methods, which have shown considerable advantage compared to currently established techniques.

Another main holdback of novel polymersome formulations is that these are typically studied in isolation and not compared to analogous systems or commercially available ones with respect to key *in vivo* characteristics such as circulation times, specificity of uptake, potency, and delivery of the protein or drug. Such comparisons are, however, needed to verify whether the extra synthetic efforts needed for polymersomes are warranted and actually generate better and prolonged therapeutic delivery with respect to established methods. A further critical point, which differentiates polymersomes from most liposomes, is their end-of-life fate once the cargo has been delivered and the polymersome now needs to be excreted from the body. While liposomes are typically made from naturally available lipids, which can be easily degraded through established mechanisms in the body, similar pathways are not as well established for polymers, and thus polymersome assemblies. Moreover, these polymersomes often incorporate non-degradable polymeric backbones, which can have detrimental side effects *in vivo*, such as accumulation in tissues and organs. As long as the exact fate of polymersomes are unknown, such formulations are more difficult to be approved by regulatory agencies.

Apart from the outlined difficulties with polymersomes, the combined research efforts of the last thirty years have provided a solid foundation on which the polymersome field now stands. The ability to functionalise polymers and fully assembled polymersomes with any moiety of choice has led to a library of polymersomes being available. The first comparative studies of polymersome formulations and established therapeutics are also being published which allows to identify further advantages of polymersomes during drug delivery in addition to their inherent structural factors mentioned above. It is becoming clear that polymersomes possess attractive advantages in therapeutic drug delivery, but that the intricate workings of the human body call for further fundamental understanding of how these can be best valorised. One of the best examples is the functionalisation of polymersomes with cell-specific receptors: the synthesis of such polymersomes is now trivial but the prediction and exclusive localisation of these polymersomes in one type of tissue or cell is still very challenging. It should be noted, however, that this is an issue pertinent to any drug delivery vehicle.

The development of new biodegradable polymers represents an active area of research and related discoveries and a growing understanding of how these polymers degrade *in vivo* will help polymersome formulations to rival liposomes during their end-of-life. Yet again, the versatility of the polymer backbone enables an exact tuning of the degradation of the polymersome assembly to the targeted application. Furthermore, the controlled or programmed disassembly of polymersome can also work in favour of the therapeutic effect. All of the above points towards the need to critically analyse the *status quo* in polymersome research, and to identify the most relevant and promising proceedings and processes in order to accelerate the access of polymersomes to the clinic.

## Author contributions

M. G. G., J. P. W., J. R.: writing – original draft, review & editing; visualisation. C. W.: writing – review & editing, supervision, funding acquisition. P. B. V. S.: conceptualisation, writing – original draft, review & editing; visualisation; supervision, funding acquisition. N. B.: conceptualisation, writing – review & editing, supervision, funding acquisition. All authors read and gave consent to the final version of the manuscript.

## Conflicts of interest

The authors have no conflict of interest.

## Supplementary Material
